# Morals Matter in Economic Games

**DOI:** 10.1371/journal.pone.0081558

**Published:** 2013-12-16

**Authors:** Felix C. Brodbeck, Katharina G. Kugler, Julia A. M. Reif, Markus A. Maier

**Affiliations:** Ludwig-Maximilians-Universitaet Muenchen, Munich, Germany; George Mason University / Krasnow Institute for Advanced Study, United States of America

## Abstract

Contrary to predictions from Expected Utility Theory and Game Theory, when making economic decisions in interpersonal situations, people take the interest of others into account and express various forms of solidarity, even in one-shot interactions with anonymous strangers. Research in other-regarding behavior is dominated by behavioral economical and evolutionary biological approaches. Psychological theory building, which addresses mental processes underlying other-regarding behavior, is rare. Based on Relational Models Theory (RMT, [1]) and Relationship Regulation Theory (RRT, [2]) it is proposed that moral motives influence individuals’ decision behavior in interpersonal situations via conscious and unconscious (automatic) processes. To test our propositions we developed the ‘Dyadic Solidarity Game’ and its solitary equivalent, the ‘Self-Insurance Game’. Four experiments, in which the moral motives “Unity” and “Proportionality” were manipulated, support the propositions made. First, it was shown that consciously activated moral motives (via framing of the overall goal of the experiment) and unconsciously activated moral motives (via subliminal priming) influence other-regarding behavior. Second, this influence was only found in interpersonal, not in solitary situations. Third, by combining the analyses of the two experimental games the extent to which participants apply the Golden Rule (“treat others how you wish to be treated”) could be established. Individuals with a “Unity” motive treated others like themselves, whereas individuals with a “Proportionality” motive gave others less then they gave themselves. The four experiments not only support the assumption *that* morals matter in economic games, they also deliver new insights in *how* morals matter in economic decision making.

## Introduction

In neoclassical economic theories about decision making humans are conceived as self-interested, rational utility maximizers, who behave accordingly when making decisions in interpersonal situations. The latter is modeled by game theory [3] (for a review see 4). However, ample empirical evidence exists, from evolutionary biology (e.g., 5), behavioral economics [6], and more recently also from neurobiology and neuro-economics (e.g., 7,8), which demonstrates that people take the interest of others into account, are sensitive to norms of cooperation and fairness, and express various forms of solidarity with others when making decisions in interpersonal situations like economic games, even when anonymous strangers are involved and when interaction is singular (i.e., one-shot games).

A common subject of interest across the disciplines cited is referred to as *other-regarding behavior*, that is, the apparent concern of agents for outcomes and behaviors affecting others, expressed behaviorally, for example, by giving others a share of windfall gains in the Dictator Game [9] or in the Solidarity Game [10], by contributing to a public pool or by paying to punish defectors in the Public Good Game (e.g., 11–13). Across all above cited disciplines, psychological processes are commonly assumed, or post hoc concluded, to underlie the activation and regulation of other-regarding behavior (e.g., altruistic motives, strategic considerations of reputation building, social norms for cooperation and fairness). However, there are few attempts to actually integrate psychological theorizing in the domain of other-regarding behavior (for an exception see 14,15) and experimental studies investigating *psychological mechanisms*, which underlie the enactment of other-regarding behavior, are rare (for exemptions see 16,17). On a side note it should be mentioned that Bazerman and Malhotra [18] go as far as arguing that psychological findings are widely neglected by economic researchers as well as by economic and organizational policy makers. In their review of common myths in economic decision making research, the authors conclude that basic assumptions which are commonly shared among economic researchers are myths according to well established psychological findings, such as the assumptions that individuals have stable and consistent preferences, know their preferences, or behaviorally pursue known preferences with volition. Most notable is the myth that “credible empirical evidence consists of outcome data, not of mechanism data [which] ignores the fact that psychological mechanisms predict behavior and outcomes” (p. 278).

This state of affairs leaves important questions unanswered. What are the psychological antecedents and mechanisms underlying other-regarding behavior in interpersonal decision making, alongside evolutionary predisposition, neurobiological hardwiring, and rational choice paradigmatic modeling? How is other-regarding behavior psychologically triggered and regulated in interpersonal situations of decision making? And, of what nature are the underlying psychological processes, are they automatic or conscious, or both? Our research was inspired by this lack of psychological theory building in the area of other-regarding behavior, which is currently dominated by economical and biological approaches. 

We identified two psychological theories, notably Relationship Regulation Theory (RRT, [2]), and its precursor, Relational Models Theory (RMT, [1]), which address psychological mechanisms underlying peoples’ constructions of social relationships, and how these influence the formation and enactment of other-regarding behavior. In a series of four experiments (plus two pilot experiments) we implemented experimental paradigms, based on the Solidarity Game [10], and tested three propositions, derived from RRT and RMT, about the activation and regulation of other-regarding behavior in one-shot economic decision making games involving strangers.

In the following the current state of theory building about antecedents of other-regarding behavior and their impact on decision making, exemplified in economic decision making games, is outlined. The discussion covers theoretical developments from evolutionary biology, neurobiology, and behavioral economics (for current reviews of these fields see 5,6,8,19 and delineates the scope for psychological theorizing. Based on Rai and Fiske’s RRT [2], Fiske’s RMT [1], and Haidt’s synthesis of moral psychology [14,15], we develop our theorizing about psychological variables regulating other-regarding behavior. Thereby, we present three propositions, which address the questions raised above, and test them in a series of experiments. 

### Cooperation through Self-Interest and Beyond

Early evolutionary biology informs us that self-interest of genes can result in altruism of people via *kin selection* [20] and *reciprocal altruism* [21]. While an altruistic act is costly for the giver but beneficial to the receiver, reciprocal altruism, in its original sense [22], has been defined as an exchange of altruistic acts between the same two individuals, so that both obtain a net benefit. The concept of reciprocal altruism was carried on – with a slight change in connotation, from altruism to cooperation – by behavioral economists and evolutionary biologists under the term *direct reciprocity* (“You scratch my back, and I’ll scratch yours”). It describes how individual self-interest can result in cooperation among people who are strangers to each other following the principle “if I cooperate now, you may cooperate later” ([5], p. 1560). 

According to the perspectives described above peoples’ other-regarding behavior is perceived to stem from a biological predisposition to maximize one’s own benefit and from strategic and rational considerations related to reputation building in order to pursue one’s self-interest during *repeated* interactions with the same other. While direct reciprocity is modeled in behavioral economics via game theory and its derivatives, forms of so called *indirect reciprocity* are harder to explain. As Nowak and Sigmund [23] note, “it is harder to make sense of the principle ‘You scratch my back and I’ll scratch someone else’s’ or ‘I scratch your back and someone else will scratch mine’“ (p. 1291). The first route of indirect reciprocity can be based on reputation building through ‘gossip’ [24] and a person’s conscious and rational consideration of its effects on himself or herself (i.e., “presumably I will not get my back scratched if it becomes known that I never scratch anybody else’s”). However, the second route puzzles researchers, because it requires answers to the question of “why should anybody care about what I did to a third party?” ([23], p. 1291). 

Gintis [25] presented an answer to this question by introducing the concept of *strong reciprocity* as a human trait, which operates beyond self-interest and strategic considerations for reputation building. It is defined as a predisposition to cooperate with others, and it results, for example, in kind behavior to those who are being kind (strong positive reciprocity), or punishment behavior when norms of cooperation and fairness are violated (strong negative reciprocity). Fehr, Fischbacher, and Gächter [26] point out that the “essential feature of strong reciprocity is a willingness to sacrifice resources for rewarding fair and punishing unfair behavior even if this is costly and provides neither present nor future material rewards for the reciprocator” (p. 3). 

Strong reciprocity is also shown during *one-shot interaction* among strangers and when not directly involved, as in so called *third party punishment* or *reward* [27]. People seem to derive direct satisfaction, with respective neurobiological correlates, from punishment of norm violations [27] and they experience an inner “warm glow”, again with respective neurobiological correlates, from complying with normative prescriptions, for example, by giving to charity or public goods, even when it is a mandatory deduction like a tax [28]. Furthermore, research shows that strong reciprocity operates across many cultures, even when investigating non-student populations in non-industrialized societies or communities [13]. 

Some researchers have argued that strong reciprocity might be unique to humans, speaking to a self-regarding nature of animals, including primates like chimpanzees (e.g., 29–31). However, by raising the question of how strong reciprocity might have been naturally evolved, Brosnan and de Waal [32,33] present empirical evidence that non-human primates (capuchin monkeys, chimpanzees) are more interested in their relative benefit in comparison with a conspecific partner, than in absolute benefits. These studies not only provide a beginning for the exploration of a ‘sense of fairness’ in nonhuman species, they also align with recent theories about the evolution of human cooperation and morality in general [19] and strong reciprocity in response to another’s pain, need, or distress in particular (i.e., “directed altruism” [34]), which both support Gintis’ [25] trait concept of strong reciprocity as a predisposition of humans to cooperate with others.

### How Morals Come into Play

Research from evolutionary biology and behavioral economics suggests that strong reciprocity is a powerful mechanism underlying cooperation among strangers, even in one-shot interactions. On the one hand, from evolutionary biology, which is guided by the aim to explain the emergence of human societies, the assumption is drawn that strong reciprocity is connected with the *origins* of pro-social motivations and moral norms (e.g., 19,23,35). On the other hand, from research in behavioral economics the assumption is drawn that strong reciprocity is a powerful device for the *enforcement* of moral norms and pro-social motivations (e.g., for sharing of resources and risk, for collective action) in interpersonal situations of economic decision making (cf. 26). Nevertheless, for understanding *how* other-regarding behavior is regulated within the individual human mind, the approaches from evolutionary biology and behavioral economics need to be complemented by theoretical approaches which directly address the *psychological* (i.e., cognitive, motivational, emotional) *mechanisms* underlying the individual regulation of other-regarding behavior via social motivations and moral norms. Recently presented theories of moral psychology (e.g., 1,2,14) appear a perfect fit for the study of the roles and functioning of moral norms and social motivations assumed to shape the expression of other-regarding behavior in interpersonal situations of decision making. 

The capacity for internalizing moral norms and developing social motivations seems to be a human universal (e.g., 36). What makes things complicated is that the structure and content of moral norms are culture specific (e.g., 37), and their enactment appears to be strongly situation specific [1,2,15]. This makes it difficult to develop a universal psychological theory about antecedents and mechanisms underlying the formation and regulation of peoples’ other-regarding behavior. Recent developments in moral psychology appear helpful to address these difficulties because they open new avenues of research about other-regarding behavior and the achievement of cooperation among strangers. One such approach is proposed by Haidt [15] in his ‘new synthesis in moral psychology’, and another one by Rai and Fiske [2] who propose that ‘moral psychology is relationship regulation’, thereby presenting Relationship Regulation Theory (RRT, [2]), which overlaps strongly with its precursor Relational Models Theory (RMT, [1]). 

In the following paragraphs we derive theoretical propositions from Haidt’s [15] synthesis, Fiske’s RRT [1], and Rai and Fiske’s RMT [2], thereby developing step by step our psychological theorizing. Thereafter, each proposition is made subject to repeated experimental testing in a series of one-shot economic games involving strangers.

### Moral Motives Determine Other-regarding Behavior

Rai and Fiske [2] argue that understanding the universal nature of morality while also acknowledging the worldwide disagreement about moral considerations requires the investigation of culturally universal kinds of *relationship regulation* people employ to identify moral obligations and prohibitions in their respective social contexts. The authors propose four universal and distinct moral motives which correspond to the four relational models formulated by RMT [1]. Each of the four basic moral motives comprises the relevant set of moral obligations entailed in the corresponding relational models. Rai and Fiske [2] use the term “motive” to indicate that RRT provides not only explanations for moral evaluations but also for the motivational forces to pursue the behaviors required to regulate and sustain social relationships respectively. The moral motives formulated by RRT are directed toward *Unity*, *Hierarchy*, *Equality*, and *Proportionality*. When relevant social relationships are absent, not activated or not attended to, no kind of moral motive is salient (i.e., *Null* morality) which leads to moral indifference, as apparent, for example, in dehumanization or moral disengagement [1,38].

*Unity* is the moral motive embedded in Communal Sharing (CS) relational models and serves as other-regarding motivation to care for and support in-group members by avoiding threats and providing aid based on need or empathic compassion. *Hierarchy* is the moral motive embedded in Authority Ranking (AR) relational models and serves as other-regarding motivation for creating and maintaining linear ranking in social groups (e.g., subordinates are motivated to respect and obey the will of superiors, who in turn are motivated to lead and protect subordinates). *Equality* is the moral motive embedded in Equality Matching (EM) relational models and serves as other-regarding motivation for enforcing equal balance and one-to-one balanced in-kind reciprocity in social relations (e.g., “scratch my back and I will scratch yours” or “pursuing eye-for-an-eye forms of revenge” [2]). *Proportionality* is the moral motive embedded in Market Pricing (MP) relational models and serves as other-regarding motivation for judgments to be based on a utilitarian calculus of costs and benefits and rewards and punishments proportional to relative merit or opportunity. The relational models, which form the base of moral motives, are distinct categories and usually people apply one dominating model or a combination of models when interacting in social contexts [1]. 

These constructions of relationship regulation, with their embedded moral motives, are universal, but cultures and individuals may differ in which contexts or situations respective motives are activated and how they are implemented and enacted [2,39]. Unlike other theories of moral behavior [15,40,41] RRT predicts that any action (even apparent violence, unequal treatment or apparently strong forms of selfishness) can be perceived as morally correct depending on how the relevant interpersonal relationships are constructed and what moral motives are employed by an individual in a given social context. This means, for example, that fairness does not necessarily imply impartiality and equal treatment, as it appears to be assumed by Haidt [15] or Turiel [41]. In contrast, RRT predicts that equal treatment and impartiality will only be judged as fair by a person if that person employs an Equality motive. Equal treatment, as for example, in the sharing of resources or responsibilities, would be morally prohibited when a person is employing a Hierarchy motive, whereby superiors are entitled to greater shares and responsibilities (e.g., [42], discussed in more detail below), or a Proportionality motive, whereby shares and responsibilities are to be distributed by relative merit or contribution, or a Unity motive, whereby in-group members feel entitled to preferential treatment over out-group members. Note that the often found incommensurability of different moral motives does not imply that there are no immoral motives. Individuals can violate the requirements of moral motives within their respective social contexts (e.g., due to temptations or shortsighted self-interest). Such action is considered a genuine moral violation in RRT.

The extent to which an actor shows a particular other-regarding behavior (e.g., in the form of solidarity, altruism, pure self-interest, or third party punishment) in an economic decision making game is shaped by the actor’s perception and definition of the situation, which according to RRT and RMT is formed by basically four kinds of relational models (CS, EM, AR, MP) with their respective moral motives (Unity, Equality, Hierarchy, Proportionality) embedded in them. Depending on the moral motive predominantly activated, respective motivational-cognitive processes structure the actor’s subjective perception of a given interpersonal situation and evoke corresponding moral motives, which are expressed behaviorally in a given interpersonal decision making context.

**Proposition 1.** The expression of particular other-regarding behaviors in one-shot economic decision making games is determined by the kind of moral motive that is activated (or salient) within an actor’s mind.

While predictions from RMT have been explored and tested in a wide array of social situations and content domains (for reviews see 1,2, for a bibliography of relevant studies see www.rmt.ucla.edu) experimental studies about interpersonal economic decision making, employing assumptions derived from RMT are rare. The few studies currently available support the proposition that relational models, once made salient to the actor (e.g., by framing or cueing of characteristics of the situation or the agents involved) influence emotional reactions toward others, evaluations about others’ behaviors, and decision making behavior in interpersonal situations. In an experimental study about mental accounting participants accepted proposals to buy objects acquired in MP relationships (pertaining to *Proportionality* motives) as routine, whereas the same proposals in CS (*Unity*), AR (*Hierarchy*), and EM (*Equality*) relationships triggered distress and erratically high dollar valuations [43]. In three experiments about consumer evaluations of consumer brands and their practiced type of customer relations management (CS-*Unity* versus a mixture of EM- *Equality* and MP-*Proportionality* motives), Aggarwal [44] provides support for the assumption that relational models influence brand evaluations by customers. And, in a series of five experiments, Fiddick and Cummins [42] show that establishing AR (*Hierarchy*) norms (in the sense of “noblesse oblige”) predicts behavioral tolerance of free riding (of ‘subordinates’) when a high-ranking perspective is adopted.

To the best of our knowledge, no experiment about other-regarding behavior in economic decision games has been published (yet), which explicitly refers to RRT. However, RMT and RRT strongly overlap conceptually, in that moral evaluations, as specified in RMT, are intertwined with motivational forces to pursue the behaviors required to regulate and sustain social relationships accordingly, as specified in RRT. Thus, findings reported with respect to predictions derived from RMT, pertaining to the CS, AR, EM, and MP relational models are likely to be of high relevance for predictions derived from RRT, pertaining to *Unity*, *Hierarchy*, *Equality*, and *Proportionality* moral motives respectively. 

### Other-regarding Behavior Needs no Rational Footing

Haidt [14,15] draws on Zajonc’s [45] dictum, “preferences need no inferences” and the works from Bargh and Chartrand [46] and Fazio, Sanbonmatsu, Powell, and Kardes [47], when arguing that a useful distinction in moral psychology is between “moral intuition” and “moral reasoning”. Moral intuition refers to an automatic and often affect-laden process, as a result of which an evaluative feeling (e.g., good or bad, prefer or reject) appears in consciousness. In contrast, moral reasoning is a controlled and often a less affective conscious process by which information about relationships and peoples’ actions is transformed into a moral judgment or decision. Furthermore, a particular sequence of events is suggested, such that moral reasoning is usually a post-hoc process in which people search for evidence to support (less often to disconfirm) their initial intuitive reaction (i.e., the ‘intuitive primacy principle’ [14,15]). Empirical support for the intuitive primacy principle is seen in, for example, neurobiological evidence demonstrating people’s nearly instant implicit reactions to moral violations (e.g., 48), the high predictive power of affective reactions for moral judgments and behaviors (e.g., 49), and further evidence from cognitive psychology, showing a disparity of ‘feeling that something is wrong’, while not being able to say ‘why it feels wrong’ [50]. 

On the basis of these considerations about moral intuition and moral reasoning, we argue, that for situations in which relationship regulation is required, as for example in economic decision games, both types of processes, automatic and conscious, are involved with the activation of particular relational models and respective moral motives, and the expression of appropriate other-regarding behavior. (Whether this is the case in an order of sequence, as suggested by Haidt [15], or inextricably mingled together, as suggested by Knobe [51], or in another form, such as described in dual process models [52], where the two types of processes interact at certain stages in their deployment, must be left open in the present study.) Rai and Fiske [2] touch the distinction between moral intuition and moral reasoning only briefly, to make the point, that both are not based on asocial principles of right actions, as is proposed by Hauser [40] or Mikhail [53], or on concerns with “purity”, as is proposed by Haidt [15]. Instead, the authors *define* moral intuition and moral reasoning by the particular types of relational models and respective moral motives that are evoked (or salient) in an individual’s mind when confronted with a particular interpersonal situation of decision making. Although not explicitly formulated as part of RRT, from the earlier theoretical and empirical work about RMT, it can be inferred that relational models function consciously and unconsciously (automatically), which includes unconscious processes of prototype formation and automatic categorization [54,55]. We thus find it plausible to assume that the unconscious (or automatic) activation of a particular kind of relational model (RMT) also results in an unconscious activation of respective moral motives (RRT) which are expressed in accordant other-regarding behaviors in interpersonal situations of decision making. 

**Proposition 2**. The expression of particular other-regarding behaviors in one-shot economic decision making games is determined by the kind of moral motive that is - consciously or unconsciously - activated (or salient) within an actor’s mind.

### Effects of Moral Motives are Confined to Interpersonal Situations

While abstract decisional problems, with no personal ramifications for others, are performed in the manner an idealized scientist or judge would perform them, moral problem solving is designed to work for *social* doing in interpersonal situations (‘*moral* thinking is for *social* doing’ [15], p. 999). This is in line with the perspective taken by Rai and Fiske [2] in RRT. According to RRT the psychological processes, underlying the four fundamental relational models and respective moral motives, serve the regulation of relationships, which binds them to *interpersonal* situations of decision making. In *solitary* situations of decision making, no other party is apparently involved who is (or might be) directly affected by the actor’s decision behavior - except the actor himself or herself. Thus, relationship regulation is not required (whereas self-regulation is) and moral motives, once (made) salient in a person’s mind, should not affect decision behavior. Thus, when activated in *solitary* situations of economic decision making, moral motives should not have a noteworthy impact on a person’s decision behavior. 

**Proposition 3.** Economic decision making behavior remains unaffected by the kind of moral motive, which is - consciously or unconsciously - activated in a *solitary* situation. 

To summarize, we conducted four experiments, each comparing the behavioral effects of two different moral motives according to RRT (Unity versus Proportionality). Experiments 1 and 2 address the first two predictions that the expression of other-regarding behavior in a one-shot economic decision making game is determined by the kind of moral motive (Unity versus Proportionality) made salient to the actor, by explicitly framing the whole experimental situation accordingly (Experiment 1, conscious activation), and by subliminally priming the two different moral motives in a precursory part of the experiment (Experiment 2, unconscious activation). To test the prediction that moral motives affect economic decision making in an interpersonal situation but not in a *solitary* situation, and to replicate the results from the first two experiments, two further experiments (Experiments 3 and 4) employing the same moral motives (Unity versus Proportionality) and types of activation (framing versus subliminal priming) were conducted. More specifically, in Experiments 3 and 4 an interpersonal situation and a *solitary* situation (with a concordant decision task) of economic decision making were compared. In order to pre-test the newly developed decision game paradigms for our experiments and to establish control conditions, two pilot experiments, with no manipulation of moral motives, one with an interpersonal and one with a concordant *solitary* situation of economic decision making, were conducted besides the main series of four experiments.

## Experiment 1

In Experiment 1, we used a novel game paradigm, which is a modified 2-player version of the originally 3-player Solidarity Game (SG), first presented by Selten and Ockenfels [10]. We termed it Dyadic Solidarity Game (DSG; for a description see File S1, Appendix A). Selten and Ockenfels’ [10] SG is well established in behavioral economics and it is known to allow for the expression of more or less (or no) solidarity in other-regarding behavior. SG was shown to be robust against instructors’ cues [56] and sensitive to differences in cultural norms [57]. The possible individual decision making behaviors in Selten and Ockenfels’ [10] SG and our modified DSG range from expressions of solidarity, in the sense that a person helps another person to a certain extent in the form of unconditional gift giving, to pure self-interest driven behavior, in the sense of maximizing one’s personal utility by not giving (much or anything) to the other person. 

Selten and Ockenfels [10] define solidarity as gifts that are made but not (necessarily) reciprocated. The authors describe solidarity as a ‘subtle form of reciprocity’, which is different from ‘giving *after* one has received’. In both, Selten and Ockenfels’ [10] SG and the here presented DSG, a gift can be made to another person, who presumably, if one were in need oneself, would make a gift to oneself. Both are one-shot games with participants being anonymous to each other, with a fixed 2/3 chance of winning and a 1/3 chance of losing determinable financial resources. Thus in both games there are two forms of risks to consider: (1) a *probabilistic risk*, which does call for rational computation and respective decision behavior, and a (2) *relational risk* (or ‘moral hazard’, cf. [58]) with the option to more or less (or not at all) mitigate the risk of total loss for *the other* person who might or might not be willing to mitigate one’s own risk of total loss. In both types of games, participants can decide to show a certain extent of solidarity behavior towards the other person and a certain extent of maximizing their personal expected utility. According to expected utility theory the personal utility is maximized (in SG and DSG) when nothing is given to the other person (for the case of losing). Considerations of relational risk call for relational or moral information processing, and thus, according to our theorizing should be influenced by the kind of moral motive that is (made) salient in a person’s mind.

All respects in which DSG differs from Selten and Ockenfels’ [10] SG are neither beneficial to the affordances of our study (e.g., SG is a complex three person game, DSG is a simple two person game), nor are they necessary for testing our predictions (for further details about similarities and differences between SG and DSG see File S1, Appendix A). However, one essential difference needs to be pointed out, because it was our major reason for modifying the SG for the present series of studies: In DSG a person’s gift giving is *fully unconditional*. In the DSG, which involves two players, each player decides to allocate a certain amount of money, which is given to the other player in case this other player is losing. In case the other player is winning this amount is not returned but withhold by the Experimenter. Thus the *gift giving* is unconditional (and not conditional upon the other player losing) and the probabilistic risk is held constant, which allows the targeting of relational risk considerations by inducing moral motives. In contrast, in SG, which involves three players, *gift receiving* is not only conditional upon oneself losing (as in DSG) but also on one or two other participants winning. If all three players lose, there is no gift reception in SG. Furthermore, the amount of money, which is assigned to be a gift to the other players, is returned if the other players do not lose (i.e., if all players win). This may be driving some of the results reported by Selten and Ockenfels [10], as was argued by Charness and Genicot [59]. The apparent complexity of the pay-off distributions in SG appears to have confused a considerable proportion of participants [10]. These, potentially confusing, conditions are excluded in the newly developed DSG where two persons engage in one-shot interpersonal decision making in a dyad. Both participants receive the same amount of money to their disposal. Each person can win up to the full amount with a probability of 2/3 or lose with a probability of 1/3. Before the lottery draw, each person decides whether and how much money he/she wants to put aside, which will be given to the other person in the case of losing. Hence participants can divide their financial resources in two partial amounts (Amount A and Amount B). Each person receives Amount A for his/herself in case of winning. In case of losing, each person receives the Amount B put aside by the respective other person (for more details on the DSG see File S1, Appendix A). 

In order to empirically establish a baseline (with no manipulation of moral motives) and to test for empirical equivalence with the previously published SG outcomes, the DSG paradigm was pre-tested in a DSG Pilot Experiment (see File S1, Appendix A). Our intention was to implement a one-shot interpersonal decision game, which allows for the above described considerations and expressions of other-regarding behavior in a simple and straightforward way. In our view and according to the results from the Pilot Experiment, which are highly comparable to respective SG outcomes, this is the case in the newly developed DSG paradigm. 

The purpose of Experiment 1 was to test the differential behavioral effects of two different moral motives in economic decision making, as stated in our first proposition. As mentioned before, the behavior in DSG can vary from decisions that represent the maximum of a cost-benefit analysis and no solidarity to decisions representing a worse individual payoff but higher levels of solidarity (in the form of unconditional gift giving). Therefore Unity and Proportionality moral motives (cf. 1,2) were selected for experimental comparison. Regarding economic decisions - in other words the exchange and distribution of benefits and risks - Unity moral motives should be associated with a cooperative use of resources and risk sharing, resulting in more solidarity in other-regarding behavior, whereas Proportionality moral motives should be associated with a use of resources and risk sharing in line with individual expected utilities, resulting in less solidarity apparent in other-regarding behavior. Based on those distinct characteristics of the respective moral motives and accordingly different considerations of relational risks, we hypothesize the following: 

**Hypothesis 1.** Individuals in a Unity condition show more solidarity behavior by giving a higher Amount B to the other person than individuals in a Proportionality condition.

### Method

#### Participants

Participants were invited to a laboratory in the Department of Psychology of the Ludwig-Maximilians-Universitaet Muenchen, Munich, Germany. In total 75 individuals from the University participated in Experiment 1 (sex: 57% female; age: *M* = 24.97 years, *SD* = 4.48 years). Participants received a bar of chocolate in addition to the game’s payoff. 

The experiment and its consent procedure were approved by the Research Ethics Committee of the School of Psychology and Pedagogy of the Ludwig-Maximilians-Universitaet Muenchen, Munich, Germany. Information about the duration, the tasks, the payment, and the confidentiality was provided to participants prior to signing up for the experiments. By voluntarily signing up for the experiments, participants provided written consent to participate in the study. Participants were able to leave the experiment at any time without consequences. 

#### Stimuli and procedure

Participants were invited to the experiment via email and written announcements placed at various locations of the University. The invitation informed all participants that they would engage in a decision task and would receive at a minimum a chocolate bar and at a maximum 10 Euros in addition to the chocolate bar. Participants were further notified about the duration of the experiment, that their participation was voluntary, and that their answers would be treated confidential. In each session four to six participants were seated together in one room, but worked individually on a computer in a private cubical. Participants were told that they would engage in a decision task together with one other person in the room, who would remain anonymous (in fact, for practical reasons, the “other person’s” behavior was simulated by a computer). Participants were randomly assigned to one of the two conditions: Unity or Proportionality (i.e., our independent variable). The conditions differed only in the introductory statement (for full descriptions, see File S1, Appendix C), which described the purpose of the overall study, either in a Unity frame (participants were told that the study is about “common welfare in groups or in the society” and “cooperative, social behavior” is examined) or in a Proportionality frame (participants were told that the study is about “cost-benefit-optimization on markets” and “individual profit maximization”). Then the DSG decision task was explained. Participants had 10€ at their disposal and were asked to make their decision regarding the division of the 10€ in Amount A (for oneself in case a dice shows a 1, 2, 3 or 4) and Amount B (for the other person, in case a dice shows a 5 or a 6). The Amount B constitutes our dependent variable. After submitting the decision, the computer randomly determined the result of throwing a dice. Subsequently participants were informed about their payoff. In case the dice showed a 5 or 6 participants received the amount B of “the other person”. In this study the other person was simulated by a computer that determined the payoff of the participant (i.e., a number between 0 and 10). At the end of the session demographic data was collected and participants received their appropriate payoff, the chocolate bar, and a full debriefing. 

#### Data availability

The data from this study, with appropriate supporting materials and explanations, will be shared upon request. 

### Results

**Figure 1:**
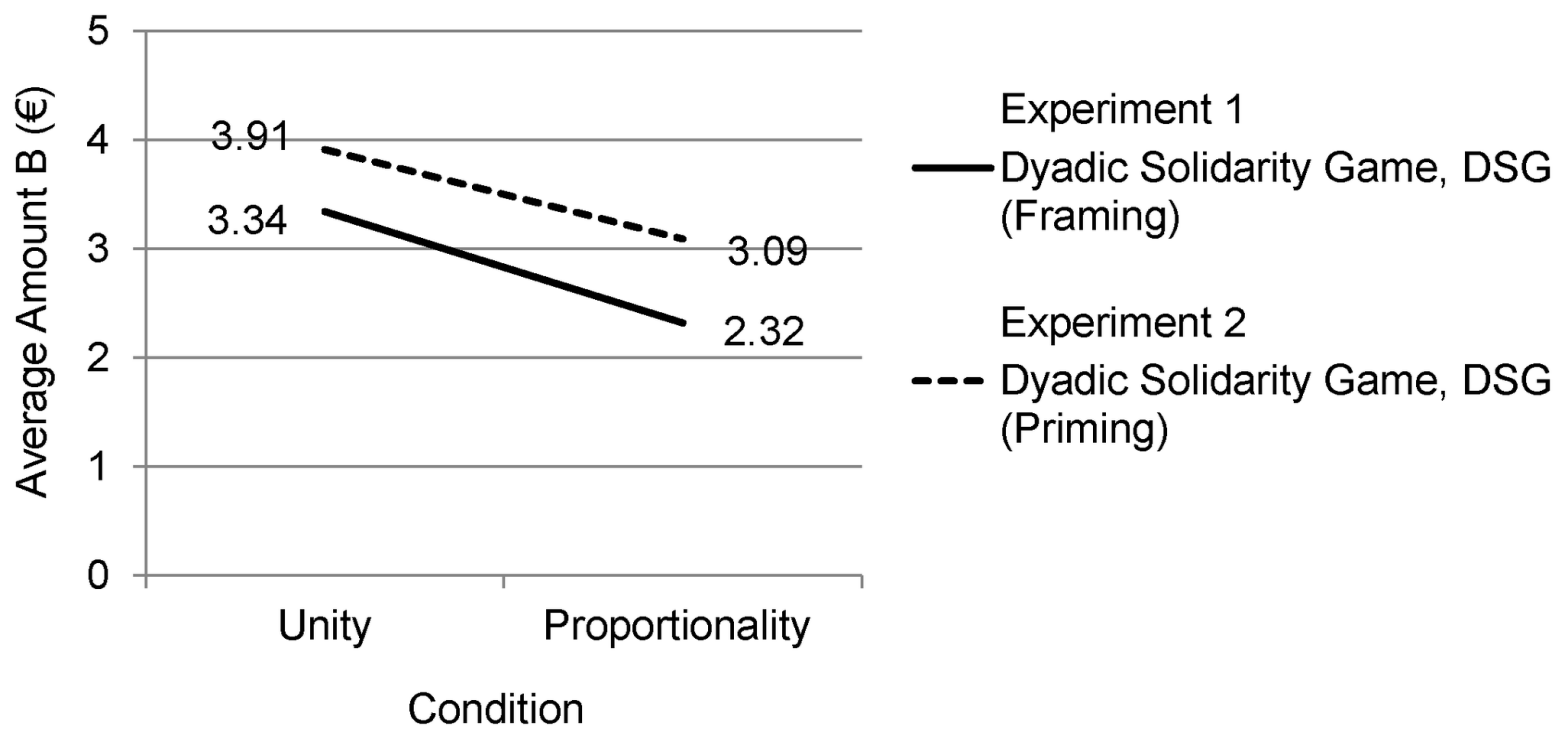
Visualization of the results of Experiments 1 and 2.

Participants from the two experimental conditions were compared regarding the unconditional gift, which they made to the other person (Amount B). In the Unity condition participants gave a higher Amount B (*M* = 3.34, *SD* = 1.46) to the other person than in the Proportionality condition (*M* = 2.32, SD = 1.51, *t*(73) = 2.97, *p* = .004, d = .69), which supports our first hypothesis. The results are presented in Table 1 and Figure 1.

**Table 1:** Descriptive Data for Experiments 1 through 4 and Pilot Experiments.

Experiment	Manipulation		Game						Show up fee	Location	Single vs. first
			DSG			SIG					
	Type	Moral motives	N	Mean	SD	N	Mean	SD			
DSG pilot	Control	No manipulation	18	2.50	1.47				4€	Department of Economics	Single
SIG pilot	Control	No manipulation				24	3.20	1.31	4€	Department of Economics	Single
1	Framing	Total	75	2.84	1.56				Chocolate	Department of Psychology	Single
		Unity	38	3.34	1.46						
		Proportionality	37	2.32	1.51						
2	Priming	Total	45	3.51	1.34				10€	Department of Psychology	First
		Unity	23	3.91	0.95						
		Proportionality	22	3.09	1.57						
3	Framing	Total	45	2.24	1.73	43	3.42	1.78	4€	Department of Economics	Single
		Unity	18	3.11	1.71	25	3.30	1.97			
		Proportionality	27	1.67	1.52	18	3.58	1.51			
4	Priming	Total	43	3.28	1.65	46	3.70	1.33	Extra credit	Department of Psychology	First
		Unity	21	3.81	1.08	24	3.58	1.38			
		Proportionality	22	2.77	1.95	22	3.82	1.30			

Note. DSG **=** Dyadic Solidarity Game. SIG = Self-Insurance Game. (Single) = the experiment was conducted as a stand-alone study; (First) = the experiment was conducted as a first experiment in a series of experiments. Means and Standard deviations show the amount of Euro (€).

Results in both conditions of Experiment 1 are inconsistent with the maximum of the expected utility, as the Amount B in each condition is significantly greater than 0 (Unity: *t*(37) = 14.14, *p* <. 001, 95% CI [2.86, 3.82]; Proportionality: *t*(36) = 9.36, *p* < .001, 95% CI [1.82, 2.82]). This means that in both conditions it is highly unlikely that individual utility maximization is the sole behaviorally impactful motive operating. Furthermore, comparisons with the mean value of Amount B (*M* = 2.50€) obtained in the control condition with no manipulations of moral motives (see DSG Pilot Experiment in File S1, Appendix A) reveals that the mean level in the Unity condition (M = 3.34€) was significantly higher (*t*(54) = 2.01, *p* = .050, d = .57), whereas the mean level in the Proportionality condition (*M* = 2.32€) was slightly below the mean level in the control condition, but did not differ significantly from it (*t*(53) = 0.42. *p* = .677, d = .12). 

## Experiment 2

The purpose of the second experiment was to test whether moral motives that are *unconsciously* induced via subliminal priming have the same effects in an interpersonal situation of economic decision making as the moral motives that were consciously induced in Experiment 1 via framing. Thus, the same two moral motives as in Experiment 1 (Unity versus Proportionality) - and the same decision making game (DSG) were used for testing our second hypothesis.

**Hypothesis 2.** Individuals in a Unity subliminal priming condition show more solidarity behavior by giving a higher Amount B to the other person than individuals in a Proportionality subliminal priming condition.

### Method

#### Participants

Experiment 2 was conducted in a laboratory in the Department of Psychology of the Ludwig-Maximilians-Universitaet Muenchen, Munich, Germany. In total 45 individuals were recruited (sex: 71% females; age: *M* = 25.57 years; *SD* = 6.78 years) from the university. The experiment was the first in a series of experiments and a 10€ show up fee was paid for participation in the entire series. In addition, participants received the amount, which they gained by engaging in the DSG. 

The experiment and its consent procedure were approved by the Research Ethics Committee of the School of Psychology and Pedagogy of the Ludwig-Maximilians-Universitaet Muenchen, Munich, Germany. Information about the duration, the tasks, the payment, and the confidentiality was provided to participants prior to signing up for the experiments. By voluntarily signing up for the experiments, participants provided written consent to participate in the study. Participants were able to leave the experiment at any time without consequences. 

#### Stimuli and procedure

Participants were invited to the experiment via email and written announcements placed at various locations in the University. They were informed in the invitation that they could engage in a series of studies, for which they would receive a minimum of 10€ and a maximum of 20€. Information about the confidentiality, the voluntariness, and the duration of the experiment was provided as well. After agreeing to participate and arriving at the laboratory, individuals were told that the series of studies started with two tasks: an “attention task” (i.e., the subliminal priming) and a “decision task” (i.e., DSG). Participants were further told that for practical reasons they would first receive the instructions for both tasks and then engage in the two tasks without interruptions. Each participant was seated alone in one room. The other person of the dyad in DSG engaged in the task with a time delay and remained anonymous. The time delay was necessary due to practical reasons, which was also communicated to the participants. The instructions for the DSG decision task, which were given to participants before the priming induction, referred to “an amount of money” without mentioning “10€” to ensure that participants did not decide on how to split their financial resources prior to the priming. After the instructions participants engaged in the so called “attention task”. More explicitly they were seated in front of a computer screen, where short sentences were presented subliminally. Participants were told to focus on the screen, follow the presentation of a fixation circle and to simply watch a letter stimulus presented after the circles. They were told they would be asked questions about the letters later during the session. Immediately afterwards participants made their decision for the decision task (i.e., DSG). They were given a sheet of paper showing 10 x 1€ coins in one row. They were asked to draw a line: on the left side of the line was the Amount A for themselves (in case a dice showed a 1, 2, 3 or 4) and on the right side was the Amount B for the other person (in case a dice showed a 5 or a 6). After the decision was made the facilitator tossed a dice and participants were given the appropriate payoff. Thereafter participants answered a short questionnaire assessing emotional states (PANAS [60], further details see below), demographic data and their recall of words (subjective awareness check of the subliminal primes), which they had identified in the attention task. Finally, participants engaged in other studies unrelated to Experiment 2 and were fully debriefed after the series of experiments ended.

The independent variable was the moral motive (Unity versus Proportionality) subliminally primed during the “attention task”. Priming is an experimental technique that is used to activate specific mental representations and to assess the behavioral consequences of this activation. It has been used to investigate automatic affective evaluations (e.g., 47), relational schemata (e.g., 61), and attachment styles (e.g., 62). While no previous studies have primed relational models, Glassman and Anderson [63] demonstrated that four-word sentences, which were presented supraliminally, activated representations of significant others. In a recent study, Shah and Kruglanski [64] successfully used short two-word sentences presented subliminally to activate specific goals in their participants. Their data indicate that even short sentences can unconsciously activate specific representations. 

In our experiment the following cues were used in order to prime participants’ salient moral motive (Unity versus Proportionality). Unity cues comprised the following three short sentences (the short sentences were translated to English, the original German sentences are provided in parentheses): we are family (Wir sind Familie), mine is also yours (Mein ist auch dein), caring for each other (Fuereinander da sein). The proportionality cues consisted of three sentences, too: what is your utility for me (Was nuetzt du mir), I want to profit (Ich will profitieren), making a deal (Einen Deal machen). In both conditions word count and number of letters were matched. I.e. in both conditions the three sentences used had in total 10 words with 46 characters each. 

In both conditions, each priming sentence was presented in the middle of the monitor twice successively: before the first presentation of a sentence a fixation circle appeared on the left side of the midpoint focusing the participant’s gaze direction to the first half of the short sentence followed by the sentence presentation; then before the second presentation of the same sentence the fixation circle appeared on the right side of the midpoint attracting the observer’s attention to the second half of the short statement. This double-presentation was chosen to support the act of “reading” from the left to the right. Each sentence presentation was masked with a forward and a backward mask. The timing of each double-presentation was as follows: right fixation circle (504 ms), forward mask (72 ms), sentence (18 ms), backward mask (72 ms), left fixation circle (504 ms), forward mask (72 ms), sentence (18 ms), backward mask (72 ms), blank screen (ISI, 504 ms). 

All three sentences were presented in this manner in random order. Before the actual priming, participants familiarized themselves with the “attention” task. For this the same presentation mode was used as in the main trials, but instead of the short priming sentences letters without meaning (e.g., Otsa kike Lpremqw) were presented. This procedure was the same in both conditions. During the practice and the priming task the letters (font: Arial; size 28) as well as the fixation circle were presented in white font on a black background. 

At the end of the experiment, after the DSG we asked the participants whether they had seen anything during the “attention task” (i.e., awareness check). Sixty-two percent of the participants indicated that they had not seen anything or named a word that had actually not been presented. Nine percent identified one or more words that were irrelevant for the priming (e.g., what), 11% identified 1 relevant word (e.g., family), 9% identified more than one relevant word, 7% correctly identified one of the three priming sentences, and 2% correctly identified two priming sentences. i.e., the majority of our sample could not consciously identify the priming content. In addition, the individual identification rate was used as an indicator of awareness of the priming stimuli. In a preliminary analysis we checked whether the amount of awareness of the priming stimuli affected our results. No biasing influence could be found (for more details see below). 

The main dependent variable was the unconditional gift (Amount B), which participants agreed to put aside for the other person for the case of loss (dice shows a 5 or a 6). In order to exclude positive versus negative affectivity as potential confounds to the primed Unity and Proportionality motives, participants’ emotional states were assessed with a short version [60] of the PANAS [65], which included a subscale for positive affect (α=.71; 5 items; 7-point scale; 1 = *low*, 7 = *high*) and negative affect (α=.75; 5 items; 7-point scale; 1 = *low*, 7 = *high*). The German translation of the items following Krohne et al. [66] was used.

#### Data availability

The data from this study, with appropriate supporting materials and explanations, will be shared upon request. 

### Results

Before conducting our main analysis, it was ensured that the two prime conditions did not induce positive or negative emotions. Participants in the Unity (*M* = 4.50.19, SD = 0.89) versus the Proportionality (*M* = 4.45, *SD* = 0.95) condition did not differ regarding positive affect (*t*(43) = 0.18, *p* = .857, d = .05). The same result was found for negative affect as participants in the Unity (M = 2.17, SD = 1.03) and the Proportionality (M = 2.16, SD = 1.02) condition showed no significant difference (*t*(43) = 0.01, *p* = .996, *d* < 0.01). Further we ruled out the possibility that the conscious recognition of words that were used in the primes weakened or reinforced the main effect of the priming (Proportionality vs. Unity). The interaction (moral motives * degree of recognition) was non-significant (β=-.04, p=.808).

In support of our Hypothesis 2 we found that participants, who were subliminally primed with Unity cues (*M* = 3.91, *SD* = 0.95) allocated a significantly (*t*(43) = 2.14, *p* = .038, d = .63) higher Amount B to the other person than participants, who were primed with Proportionality cues (*M* = 3.09, *SD* = 1.57). Results are presented in Figure 1 (Experiment 2) and Table 1. 

Results in both conditions are inconsistent with the maximum of the expected utility, as the Amount B in each condition was significantly greater than 0 (Unity: *t*(22) = 19.77, *p* <. 001, 95% CI [3.50, 4.32]; Proportionality: *t*(21) = 9.23, *p* < .001, 95% CI [2.39, 3.79]). This means that in both conditions it is highly unlikely that individual utility maximization was the sole behaviorally impactful motive. Furthermore, comparisons with the baseline of Amount B obtained in the DSG Pilot Experiment (see File S1, Appendix A) with no manipulations of moral motives (*M* = 2.50€, also see Table 1) reveals that in the Unity condition the mean (*M* = 3.91€) was significantly above the baseline (*t*(39) = 3.72, *p* <. 001, d = 1.14) whereas in the Proportionality condition (*M* = 3.09€), the mean was slightly higher than the mean obtained in the control condition, but it did not differ significantly from it (*t*(38) = 1.22, *p* = .230, d = .39). 

## Discussion of Experiment 1 and Experiment 2

In line with our hypotheses, behaviorally distinguishable responses were induced by (1) framing an experimental decision game as either part of a study about Communal Sharing norms pertaining to Unity motives or a study about Market Pricing norms pertaining to Proportionality motives (Experiment 1), and by (2) subliminally priming cues for Communal Sharing norms pertaining to Unity motives versus Market Pricing norms pertaining to Proportionality motives (Experiment 2). Experiment 2 comprises a robust replication of Experiment 1 by inducing the same moral motives of Unity and Proportionality via subliminal priming rather than conscious frames of the experimental context, while drawing on a different sample of participants, giving a different show up fee (10€ rather than a bar of chocolate), embedding the DSG in a series of experiments (rather than a single experiment), using different materials (e.g., establishing Amount A and B by using figural rather than numerical material) and tossing a real dice rather than a ‘computational’ one.

The results across both experiments support the assumption that moral motives operate consciously and unconsciously in their impact on other-regarding behavior in interpersonal economic decision making. As was predicted in our theoretical Propositions 1 and 2, individuals under a consciously or unconsciously induced Unity motive showed more solidarity behavior (i.e., giving higher amounts of unconditional gifts in DSG) than individuals under a consciously or unconsciously induced Proportionality motive. Furthermore, in the Unity conditions of both experiments the mean Amount B given was significantly above the mean obtained in the control condition in the DSG Pilot Experiment. In contrast, in the Proportionality condition of both Experiments no significantly higher Amount B as compared to the control condition was given. It appears that the ‘default’ moral motives of participants in economic decision making games are indistinguishable from Proportionality motives. However, in both experiments, as well as in the control condition, classic rational choice paradigmatic predictions (maximizing individual utility), according to which self-interest is the major, if not singular, motive that drives economic decision making in interpersonal situations (e.g., economic games), could also be rejected.

## Experiment 3

In Experiment 3 our third proposition was tested, stating that decision behavior is affected by moral motives made salient in interpersonal situations, but remains *unaffected* by moral motives which were made salient in *solitary* situations. A *solitary* situation of decision making, structurally equivalent to DSG, was developed and termed ‘Self-Insurance Game’ (SIG, for more details see File S1, Appendix B). It differs from DSG in only one respect - individuals interact with themselves and not with another person. In DSG and SIG the same *probabilistic risk* needs to be considered (i.e., 2/3 win, 1/3 lose). In DSG, as was argued above, in addition to the probabilistic risk, a relational risk needs to be considered. A relational risk is subject to relational considerations and thus should be affected by moral motives that are activated. In SIG there is no relational risk to consider, because the options to more or less (or not at all) mitigate the risk of total loss relate directly to the person itself. Participants can be 100% certain about their pay-off in case of loss. There is no “moral hazard” or “information asymmetry” (cf. 58) to consider, which includes the willingness of another person to mitigate one’s own losses (or not). We therefore hypothesize:

**Hypothesis 3.** The decision behavior in the *solitary* SIG is not influenced by the kind of moral motive made salient to a person, whereas in DSG it is affected.

A particular advantage of constructing the *solitary* SIG concordantly to DSG is that all factors potentially affecting *solitary* probabilistic risk processing can operate in both experimental conditions. From widely established research findings in behavioral economics, economic psychology, and decision sciences it is known that people display an array of probabilistic risk processing ‘biases’ in their *solitary* ‘thinking for doing’. It is ‘rationally bound’, ‘heuristic’, ‘risky’ or ‘risk averse’, to name just a few, depending on the task, the context, or personal factors (e.g., 67–71). The DSG and SIG conditions differ only with respect to the presence or absence of relational risk and the applicability of factors potentially affecting the processing of relational risk. However, there is a general possibility that forms of biased probabilistic risk processing in *solitary* decision making may interact with certain salient moral motives. Individual processing of probabilistic risk, including all kinds of potential biases, should operate in both, SIG and DSG. On the basis of Haidt’s [15] principle that ‘moral thinking is for social doing’ and the proposition derived from RRT, that moral motives are bound to interpersonal situations, we argue that activated moral motives should not impact on the more or less biased probabilistic risk processing (*for solitary doing*), but they should impact on the relational risk processing (*for social doing*). The general possibility, that different moral motives (Unity, Proportionality) *interact differently* with more or less biased probabilistic risk processing can be ruled out, when it is shown that inducing the two different moral motives does not result in different decision making behavior in SIG. In this respect, comparing allocations of resources in the SIG versus the DSG constitutes a strong experimental paradigm for testing the propositions made.

### Method

Analogous to DSG, SIG was pretested in a Pilot Experiment (i.e., SIG Pilot Experiment), which is also used as a control condition and described in File S1, Appendix B. Like in Experiment 1, the moral motives (Unity versus Proportionality) were induced explicitly by framing. A 2 × 2 (Unity versus Proportionality; SIG versus DSG) between-subject design was implemented. 

#### Participants

Participants were invited to a laboratory in the Department of Economics of the Ludwig-Maximilians-Universitaet Muenchen, Munich, Germany. A total of 89 individuals (sex: 62% female, age: *M* = 23.92 years, *SD* = 3.50 years) were recruited. Participants were paid a show-up fee of 4€ in addition to the payoff of the game. 

The experiment and its consent procedure were approved by the Research Ethics Committee of the Economics Department at the Ludwig-Maximilians-Universitaet Muenchen, Munich, Germany. Participants provided written consent to the procedures and the standards as well as participants' rights when voluntarily signing up for the panel of the laboratory. Full information about the study was provided to participants prior to the experiment and participants were able to leave the experiment at any time without consequences.

#### Stimuli and procedure

Participants were invited to the experiment via a panel, for which they had signed up previously. When signing up for the panel participants were informed about confidentiality and voluntariness as well as that they would receive a show-up fee of 4€ and an additional amount depending on the task. Information about the duration of the experiment was included in the invitation letter. Four experimental sessions were conducted; in each session one of the two games (DSG versus SIG) was played, which was determined randomly. Participants were seated in cubicles and worked on a computer. First, participants read about the purpose of the study, which was randomly framed with a Unity frame or a Proportionality frame, as in Experiment 1. The frames did not differ between the DSG and the SIG except in one detail: in the DSG participants were told that they would interact with another person during the experiment; in the SIG this notion was excluded (for details see File S1, Appendix C). Participants who engaged in the DSG were informed that they would remain anonymous to each other. Then participants received the instructions to the game, made their decision about how to divide the 10€ into Amount A and Amount B and subsequently the facilitator tossed a dice once for all participants of one session.

The dependent measure was the Amount B, which participants were willing to give to the other person in DSG, or to put aside for themselves in SIG, in case of losing (i.e., the dice showed 5 or 6). At the end participants were told their individual payoff and answered demographic questions. 

#### Data availability

The data from this study, with appropriate supporting materials and explanations, will be shared upon request.

### Results

**Figure 2:**
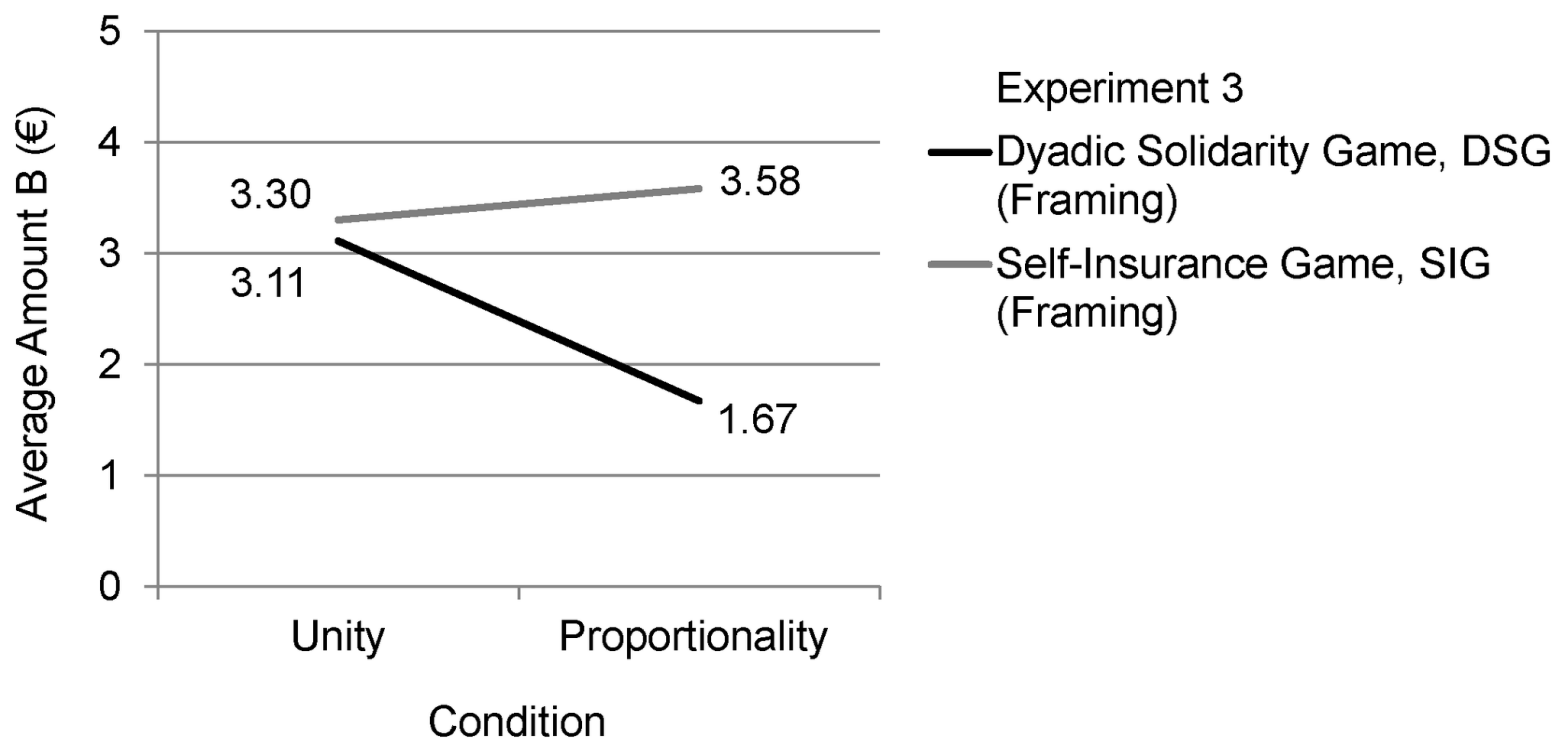
Visualization of the results of Experiment 3.

The main results are visualized in Figure 2 and descriptive data is shown in Table 1. The interaction effect between SIG versus DSG and Unity versus Proportionality conditions (decision game * moral motive) was significant (*F*(1,84) = 5.64, *p* = .021, η^2^ = .06). In the DSG condition a significant main effect for moral motives was obtained (*t*(41) = 2.97, *p* = .005, d = .89). Unity framed participants allocated a higher Amount B (unconditional gift to the other person) than Proportionality framed participants, which supports Hypothesis 1 (induced moral motives impact on other-regarding behavior) and is a premise for Hypothesis 3 (induced moral motives impact on decision behavior in DSG and not in SIG). 

In the SIG condition no significant main effect on Amount B (gift to oneself) was obtained for moral motives (*t*(41) = 0.51, *p* = .612, d = .16). Because non-significant results do not confirm equivalence between experimental groups, further analyses were undertaken using the procedure by Rogers, Howard, and Vessey [72]. It basically tests the hypothesis regarding equivalence by trying to reject an a priori defined plausible alternative hypothesis regarding a particular difference. Therefore the particular difference for the alternative hypothesis, which is aimed to be rejected, is determined first; the CI for the mean and standard deviation found in the data is determined second. If the difference of the alternative hypothesis is outside of the CI, the hypothesis of difference can be rejected and the hypothesis of equivalence can be accepted. The CI is calculated with the following formula: 

$$(M_{1}-M_{2})\pm z_{\alpha }s_{M1-M2}$$

M=mean of the experimental conditions 1 and 2

$$z_{\alpha }=\text{the}\;\text{z}\;\text{value}\;\text{for}\;\text{a}\;\text{given}\;\text{α}$$

$$s_{M1-M2}={\left\{\left[\frac{(n_{1}-1)s_{1}^{2}+(n_{2}-1)s_{2}^{2}}{n_{1}+n_{2}-2}\right]\left[\frac{1}{n_{1}}+\frac{1}{n_{2}}\right]\right\}}^{\frac{1}{2}}$$

n=number of participants in the experimental conditions 1 and 2

s= standard deviation of the experimental conditions 1 and 2

On the basis of our theorizing and empirical results from Experiment 1, it was determined, that the average Amount B in the Unity condition had to be higher than in the Proportionality condition by at least a medium effect size d ≥ .50, following Cohen [73]. Given the standard deviation of the sample the difference (Unity minus Proportionality) was computed as ≥ 0.88€. This value is not included in the 90% CI [-1.19, 0.63] and therefore the hypothesis regarding a difference between the two conditions can be rejected. Note that the 90% CI, that is, a one-sided test, was used as Rogers et al. [72] advised that “the equivalency confidence interval should be expressed at the 1 - 2α level of certainty” (p. 555). 

In summary, the results from Experiment 3 fully support Hypothesis 3, which predicts that other-regarding behavior in DSG is affected by moral motives, made salient to a person, whereas in SIG it is not affected. 

## Experiment 4

The purpose of Experiment 4 was to replicate the results of Experiment 3, this time by inducing the moral motives via subliminal priming, like in Experiment 2. Together, Experiments 3 and 4 also constitute a robust replication of the combined findings from Experiments 1 and 2, that moral motives affect other-regarding behavior in interpersonal situations via conscious and unconscious activation. 

### Methods

Analogous to Experiment 3, the present experiment comprises a 2 × 2 between-subject design (DSG versus SIG; Unity versus Proportionality). 

#### Participants

Experiment 4 was conducted in a laboratory of the Department of Psychology of the Ludwig-Maximilians-Universitaet Muenchen, Munich, Germany. A total of 89 participants (sex: 89% female; age: *M* = 23.90 years, *SD* = 5.52 years) were recruited from the university. Analogous to Experiment 2, Experiment 4 was the first study in a series of studies, for which participants received extra credits in addition to the game’s payoff. 

The experiment and its consent procedure were approved by the Research Ethics Committee of the School of Psychology and Pedagogy of the Ludwig-Maximilians-Universitaet Muenchen, Munich, Germany. Information about the duration, the tasks, the payment, and the confidentiality was provided to participants prior to signing up for the experiments. By voluntarily signing up for the experiments, participants provided written consent to participate in the study. Participants were able to leave the experiment at any time without consequences. 

#### Stimuli and procedure

The invitation procedure for Experiment 4 was analogous to Experiment 2. In Experiment 4 - just like in Experiment 2 - participants were told that they would engage in two different tasks, an “attention task” (i.e., subliminal priming) and a “decision task” (i.e., DSG or SIG). Participants received all instructions at the beginning of the session. In case participants engaged in DSG the other person remained anonymous and was working on the task with a time delay. The time delay was necessary for practical reasons, which was also communicated to the participants. The instructions about DSG and SIG did mention “an amount of money”, but not the “10€” in order to avoid that participants made the decision before the priming activity. Then participants engaged in the attention task (subliminal priming). They focused on a screen, which subliminally showed the same sentences, which were used in Experiment 2. Next participants were given a sheet of paper showing 10 x 1€ coins in a row and were asked to make their decision by drawing a line. On the left side of the line was the Amount A (the amount of money, which they received in case the dice showed a 1, 2, 3, or 4); on the right side was the Amount B, the partition they were willing to put aside for the other person in DSG or for themselves in SIG, in case the dice showed a 5 or 6. Each decision making game was one-shot. After the decision was made the facilitator tossed the dice. Then participants answered a short questionnaire, assessing their emotional state, demographic data and the words, which they had recognized in the attention task (for more details see Experiment 2). Subsequently participants engaged in other experiments unrelated to this study and were debriefed in the very end. The experiment described in this paper was always the first in the series of studies; hence the other experimental tasks cannot have had an influence on participant’s decisions. 

The two independent variables were: the induced moral motives (Unity or Proportionality), manipulated by subliminal priming as part of the attention task, and the subsequent decision making game (DSG or SIG), in which participants made decisions about allocating their resources to Amount A and B. For a detailed description of the subliminal priming stimuli see Experiment 2, in which the exact same procedure was used. In Experiment 4, 27% of the participants did not correctly identify any of the presented words or remembered a word that was not presented; 10% identified one word or words that were irrelevant for the priming (e.g., what), 38% identified one relevant word (e.g., family), 5% identified more than one relevant word, 19% correctly identified one priming sentence, 0% identified more than one priming sentence. Again, the majority of our sample could not consciously identify the priming content. In addition, the individual identification rate was used as an indicator of awareness of the priming stimuli. In a preliminary analysis we used this variable in order to check whether the amount of awareness of the priming stimuli affected our results. No biasing influence could be found (for more details see below). 

 The dependent variable was the amount of money (Amount B), which participants agreed to put aside for the other person in DSG or for themselves in SIG in the event of losing (i.e., the dice showed a 5 or a 6). To control for positive or negative emotionality that may have been induced by priming, participants’ emotional states were assessed using a short version [60] of the PANAS [65], which includes a subscale for positive affect (α = .64 items; 7-point scale; 1 = *low*, 7 = *high*) and a subscale for negative affect (α = .77; 5 items; 7-point scale; 1 = *low*, 7 = *high*). The items were translated into German by Krohne et al. [66]. 

#### Data availability

The data from this study, with appropriate supporting materials and explanations, will be shared upon request.

### Results

Before testing the hypotheses the average PANAS scores between the two priming conditions were compared. The Unity (*M* = 5.17, *SD* = 0.85, *N* = 45) and Proportionality (*M* = 4.88, *SD* = 0.80, *N* = 44) conditions did not differ regarding the positive affect (*t*(87) = 1.67, *p* = .099, d = 0.35). Similarly, we did not find significant differences in negative affect (*t*(87) = 0.91, *p* = .367, d = 0.19) between the Unity (*M* = 1.75, *SD* = 0.89, *N* = 45) and the Proportionality (*M* = 1.60, *SD* = 0.72, *N* = 45) conditions. In addition we ruled out the possibility that the conscious recognition of words that were used in the primes weakened or reinforced the main effect of the priming (Proportionality vs. Unity). The interaction (moral motives * degree of recognition) was neither significant in the DSG (β = -.11, p = .479) nor in the SIG (β = -.12, p = .423). 

**Figure 3:**
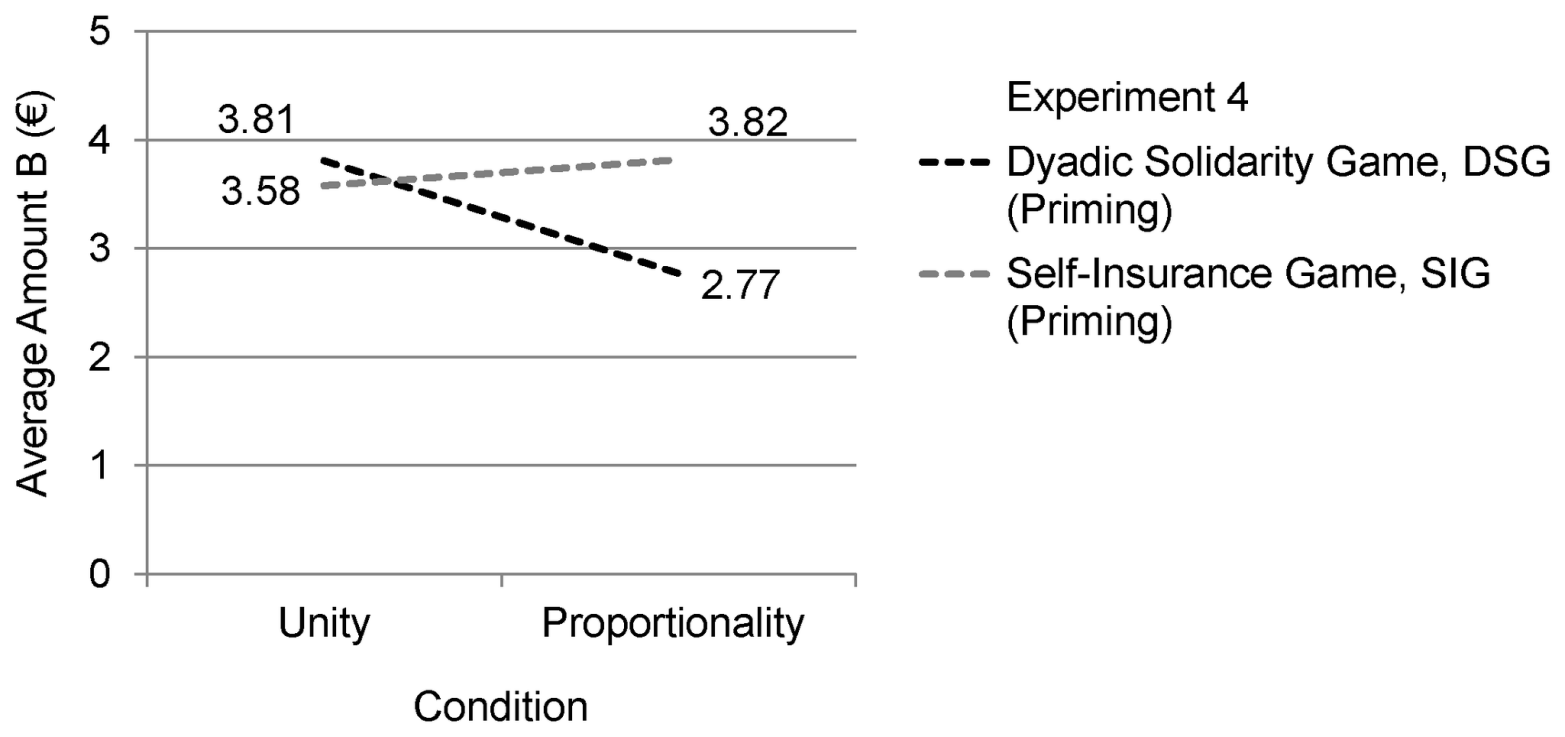
Visualization of the results of Experiment 4.

The main results of Experiment 4 are visualized in Figure 3 and descriptive data can be found in Table 1. The interaction effect between the solitary SIG versus the interpersonal DSG and the two induced moral motives (i.e., decision game * moral motive) was significant (*F*(1,85) = 4.19, *p* = .044, η^2^ = .05). Consistent with the prediction made for DSG a main effect for moral motives was obtained in DSG (*t*(43) = 2.14, *p* = .038, d = .66). Participants primed with Unity cues gave a higher Amount B to the other person than participants, who were primed with Proportionality cues. No effect of primed moral motives was found for participants who engaged in SIG (*t*(41) = .59, *p* = .556, d = .18). 

Analogous to Experiment 3 equivalence between the Unity condition and the Proportionality condition in SIG was established by using the procedure suggested by Rogers et al. [72], according to which equivalence can be assumed if a specific hypothesis of difference can be rejected. Thus a difference of d ≥ .50 (at least medium effect size; following Cohen [73]) was presumed, and given the standard deviations of the two experimental groups, this difference translates into ≥ 0.67€ (Unity minus Proportionality). This value is not included in the 90% CI [-0.88, 0.42] and therefore the hypothesis that the two experimental groups are different can be rejected on a 5% α level (for details about this analysis see Experiment 3). Consequently, our Hypothesis 3, predicting that other-regarding behavior in DSG is affected by moral motives, made salient to a person, whereas in SIG it is not, was not rejected. All results of Experiment 4, which used subliminal priming, fully replicate the respective findings from Experiment 3, where explicit framing was used.

### Under which Moral Motive does the “Golden Rule” Apply?

The SIG experimental paradigm developed for Experiments 3 and 4 allowed us to establish a plausible reference level of unconditional gift giving to oneself (i.e., self-insurance), which solely relies on probabilistic risk considerations, because the relational risk is set to zero (i.e., there is 100% certainty about what the person herself will do). Thus, with SIG we can establish behavioral responses to the question of how much participants are willing *to give themselves* in order to mitigate the probabilistic risk of total loss, when facing a probabilistic risk that is equivalent to the probabilistic risk faced in an interpersonal DSG situation (1/3). We thus used the level of gift giving ‘to oneself’ in SIG to establish the particular moral meaning attached to the level of gift giving ‘to another person’ in DSG. In other words, we tested to what extent the universal Golden Rule (“Treat others how you wish to be treated” [74]), applies under Unity versus Proportionality conditions.

As stated before, Unity moral motives imply the expectation that in a given community everyone (including oneself) should be treated equally. In contrast, Proportionality moral motives imply a focus on rewards in relation to merits, cost-benefit-analysis, and expected utilities where expectations about the other person are included. Given these characteristics of the two moral motives we explored the ‘Golden Rule’-hypothesis post hoc by using data from Experiments 3 and 4: Individuals who are subject to an induced Unity moral motive should be more likely to treat others as they treat themselves than individuals who are subject to an induced Proportionality moral motive. Thus, Unity motivated participants in DSG should give on average the same amount of money to the other person than is put aside by respective SIG participants for themselves, whereas Proportionality motivated participants should give less or nothing to the other person, which is not in line with the golden rule. 

In order to test the ‘Golden Rule’-hypothesis, we first confirmed that in the Unity condition there was no significant difference between the average Amount B in the DSG and the SIG (Experiment 3: *t*(41) = .33, *p* = .745, d = .10; Experiment 4: *t*(43) = .61, *p* = .548, d = .18). Then we conducted the significance test of equivalence according to Rogers et al. [72] (see Experiment 3 for details). Given the respective empirical standard deviations in Amount B, the difference in the Amount B between DSG and SIG in the Unity condition (DSG minus SIG) would need to be ≤ -0.92€ in Experiment 3 and ≤ -.62€ in Experiment 4, if it had at least a medium effect size in each case (d ≥ .50; following Cohen [73]). Those values are not included in the 80% CI [-0.68, 0.30] in Experiment 4 and in the 90% CI [-0.39, 0.84] in Experiment 4. The respective difference hypothesis can be rejected on a 10%-α level for Experiment 3 and on a 5%-α level for Experiment 4 (for more details regarding this analysis see Experiment 3). This means that Unity motivated participants treated others in DSG like Unity motivated participants treated themselves in SIG. In contrast Proportionality motivated participants in DSG treated the other person not on the same Amount B level as Proportionality motivated participants treated themselves in SIG. In the Proportionality condition, the allocations of solitary participants to themselves in the SIG differed significantly from the allocations of participants to others in the DSG (Experiment 3: *t*(43) = 4.16, *p* < .001, d = 1.27; Experiment 4: *t*(42) = 2.09, *p* = .042, d = .63). 

**Figure 4:**
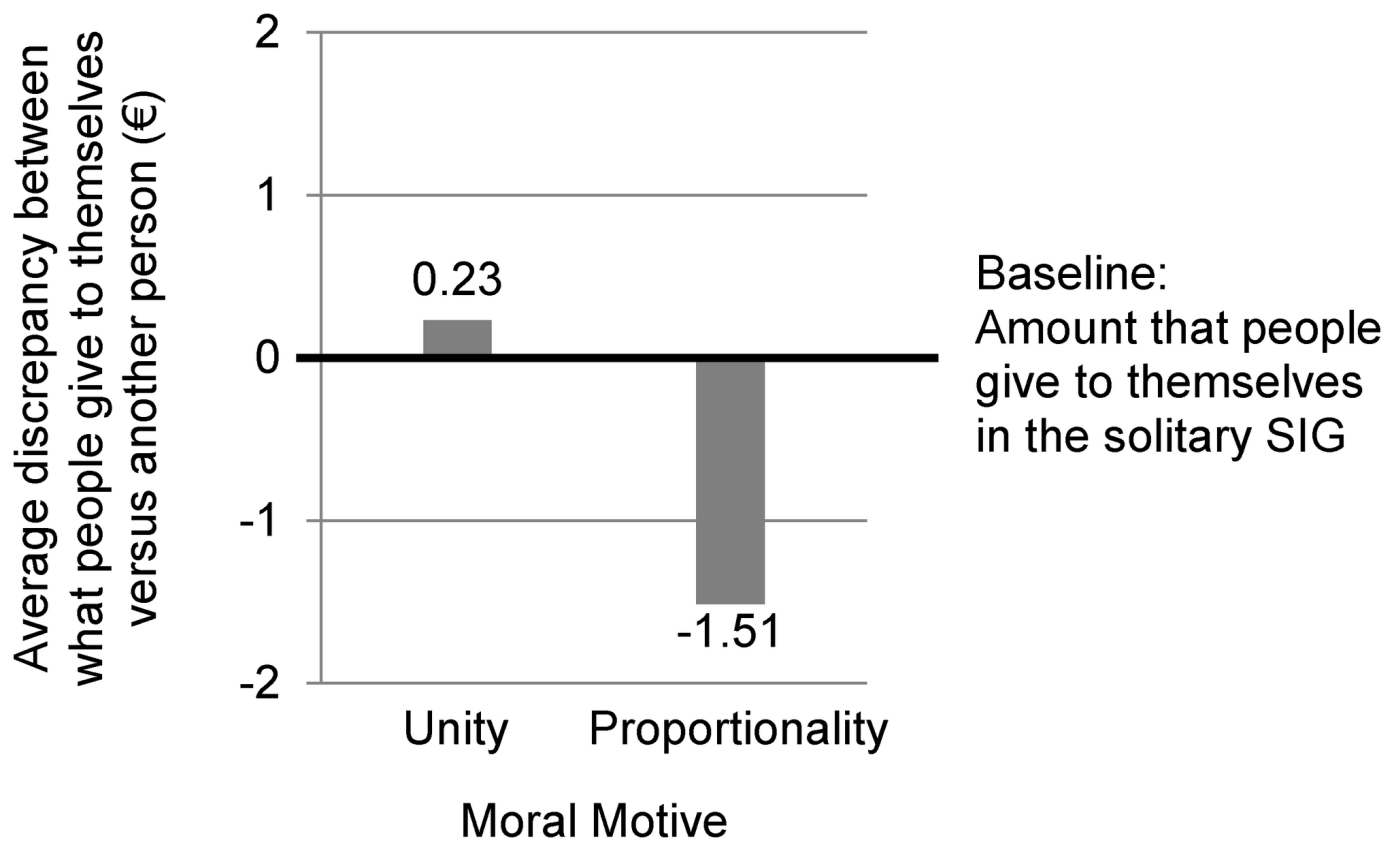
Application of the “Golden Rule” in the Unity and not in the Proportionality Condition.

In summary, the Golden Rule seems to apply to DSG participants who received a Unity moral motive treatment, either by conscious framing or by subliminal priming, and not to DSG participants who received a Proportionality treatment, whether explicitly framed or subliminally primed. For illustrative purposes Figure 4 shows the overall differences in means between the solitary SIG and the interpersonal DSG conditions in Experiments 3 (framing) and 4 (priming), which were summarized with meta-analytical procedures following Borenstein, Hedges, Higgins, and Rothstein by using the Software "Comprehensive Meta-Analysis" [75]. The results of the meta-analytic summary indicate that in the Unity condition participants give on average 0.23€ more to the other person in the DSG than they give to themselves in the SIG. In the Proportionality condition participants in the DSG give on average 1.51€ less to the other person than participants in the SIG give to themselves. 

## General Discussion

Four experiments showed that “morals matter in economic games”. The extent of other-regarding solidarity behavior in Unity conditions as compared to Proportionality conditions in the Dyadic Solidarity Game (DSG) computes to an average effect size of Cohen’s d=.70 (*z*=4.96, *p*<.001) (the average effect size was calculated with meta-analytical procedures following Borenstein, Hedges, Higgins, and Rothstein using the Software „Comprehensive Meta-Analysis”) [75]. Results repeatedly obtained in Experiments 1 through 4 support Hypotheses 1 and 2, stating that consciously and unconsciously induced moral motives impact other-regarding behavior in the DSG. In this sense, it could be shown that *strong reciprocity* behavior in one-shot economic decision games is affected by “moral reasoning” *and* “moral intuition”. Results repeatedly obtained in Experiments 3 and 4 support Hypothesis 3, stating that economic decision making behavior in DSG is significantly affected by the kind of moral motives made salient to participants, whereas in solitary situations (cf. Self-Insurance Game; SIG) it is not. It seems that relationship regulation via relational models and moral motives is confined to *interpersonal* decision situations, in which relational risks need to be considered over and above probabilistic risks - as compared to solitary situations, in which only probabilistic risks need to be considered. In this sense, it could be demonstrated that in interpersonal economic decision making games “*moral* thinking is for *social* doing” ([15], p. 999).

Our experimental results support the propositions derived from Rai and Fiske’s [2] Relationship Regulation Theory (RRT) which states that the extent to which an actor shows particular other-regarding behavior is shaped by the actor’s perception and definition of the situation, which are formed in basically four types of relational models (Communal Sharing, Authority Ranking, Equality Matching, and Market Pricing) with respective moral motives (Unity, Hierarchy, Equality, Proportionality) embedded in them. In our series of four experiments we induced and compared the behavioral effects of two of the four relational models with respective moral motives as specified in RRT (Unity versus Proportionality) by means which are *extraneous* to the proximate characteristics of the decision tasks used (i.e., by framing the experiments’ purpose accordingly and by subliminal priming immediately before the decision game). One might interpret the resulting behavioral responses to the decision situation as “spill over” effects of *extraneously* activated motives. However, as predicted on the basis of RRT, the behavioral effects of *moral* motives were shown to be specific to interpersonal (DSG) situations while not affecting decision behavior in solitary (SIG) situations. 

Future research pertaining to moral motives should directly measure the postulated moral motives as mental states and establish their mediating functions between characteristics of the proximal interpersonal decision context (e.g., particular game paradigms) employed and other-regarding behaviors expressed. To our knowledge this has not been attempted yet. 

### Unity Fosters and Proportionality Undermines the “Golden Rule”

The explanatory power of Rai and Fiske’s [2] RRT for predicting other-regarding behavior in experimental decision games could also be demonstrated by applying our newly developed game paradigm (Dyadic Solidarity Game, DSG), in combination with its solitary counterpart (Self-Insurance Game, SIG), when testing the post hoc formulated “Golden Rule”-hypothesis. It pertains to a fundamental moral principle in human societies - “treat others how you wish to be treated” [74]. In support of the “Golden Rule”-hypothesis, further analyses of our experimental data revealed that Unity motivated participants treat others in DSG equivalently to how Unity motivated participants treat themselves in SIG, whereas Proportionality motivated participants treat others in DSG significantly less favorably than Proportionality motivated participants treat themselves in SIG. Given that in the SIG no differences between Unity motivated and Proportionality motivated participants were found, we interpret the experimental results as follows: Unity moral motives foster the behavioral expression of the “Golden Rule” in one-shot decision games involving strangers, while Proportionality moral motives undermine its expression. 

Future research pertaining to moral motives could benefit from focusing on situational conditions which foster or inhibit solidarity behavior and the application of the “Golden Rule” under all four moral motives (and not only Unity and Proportionality as applied in Experiments 1 through 4) and further situational conditions under which they apply as specified by RRT. For example, Rai and Fiske [2] propose that relational models and moral motives serve the cognitive-motivational regulation of interpersonal relationships in human societies. Thus, the moral frames suggested should also apply to more complex patterns of social life, in accord with established social psychological theorizing, such as for example with respect to inter-group discrimination [76]. Unity moral motives should foster other-regarding solidarity behavior and the application of the “Golden Rule” in particular when decision game partners belong to the same ‘in-group’. In contrast, differential behavior toward ‘in-group’ and ‘out-group’ decision game partners should be less pronounced or even non-existent for Proportionality motivated participants.

### “Money” Cues Induce Proportionality Moral Motives in Decision Games

When conducting our series of experiments, we observed some systematic differences between the laboratories hosted by economics departments and by psychology departments. Money, for example, featured more prominently in economy laboratories than in psychology laboratories. Cash boxes or pay desks (for later payoff and reward) are often encountered by participants when entering the experimentation room. And for advertising experiments for participation or recruiting members for experimental panels or pools, the “money making” motive was regularly used as the major incentive to participate. In contrast, in psychology departments, in addition to the “money making” incentive, which is also used but less prominently, course credits or other non-monetary incentives were given for participation. For this reason we have conducted several replications across a variety of wider experimental context conditions. For example, we varied the show-up incentives (chocolate bar versus different amounts of money), the recruitment incentives for participants (using a pool for pay in the economic laboratory, on campus recruitment by content of the study and/or credit points), and also the use of single experiments versus omnibus experiments might have influenced the salience of “money” to participants (see Table 1, right column). 

“Money”, which is often used as a proxy for a variety of non-monetary resources and as a marker of behavioral responses in most economic game experiments, has been repeatedly reported to induce Market Pricing norms (i.e., *Proportionality moral motives according to RRT*) in various economic decision making experiments [77–79]. Vohs, Mead, and Goode [80] demonstrated that unconsciously primed money stimuli induce Market Pricing norms. Reminding of money, relative to non-money reminders, led to reduced requests for help and reduced helpfulness toward others, and participants primed with money, as compared to non-primed participants, preferred to play alone, work alone, and put more physical distance between themselves and a new acquaintance. 

According to RRT, the use of money for standard behavioral responses in economic game experiments, as well as the use of “money making” as a standard incentive for participation, and also the manifold “money” frames and primes present in economic laboratory settings, all these characteristics promote the induction of Market Pricing relational models and Proportionality moral motives with respective other-regarding behavioral outcomes. As is shown by Experiments 3 and 4 the behavioral responses in *interpersonal* decision making situations are particularly sensitive to reminders and primes of relational models and moral motives. Thus, uncontrolled and unnoticed ‘hidden’ reminders, frames and primes of money (or other morally sensitive stimuli) present in experimental game contexts are likely to distort behavioral data from decision game laboratories. 

Building on this notion we conducted an additional analysis and compared the following two conditions of our experiments: (1) DSG, conducted in the Department of Economics, using framing in order to manipulate the moral motives; (2) DSG, conducted in the Department of Psychology, using framing in order to manipulate the moral motives. Across the two frames (Unity vs. Hierarchy) we found that participants in the Department of Economics (M=2.24, SD=1.73) allocated less money to the amount B than participants in the Department of Psychology (M=2.84, SD=1.56). The results closely approached the conventional cutoff for statistical significance (t=1.94, p=.055, d=0.36). This result could potentially be explained by the fact that the money-primes in the Department of Economics induced Proportionality motives and thus participants showed less solidarity than in the Department of Psychology. However our data does not allow drawing clear conclusions and more rigorous tests of this proposition are needed. 

### Implications for the Experimental Study of Other-regarding Behavior in Decision Games

As described in the theory section, Fiddick and Cummins [42] demonstrated that inducing an Authority Ranking relational model (with *Hierarchy moral motives*) predicts an agent’s tolerance for free riding (of ‘subordinates’) better than the expected utility theory concept of self-interest does. Furthermore, the authors suggest that the common practice in behavioral economics to place participants of equal social status and no prior history in anonymous interactions fosters Equality Matching relational models (with *Equality moral motives*). This might have happened in our experiments as well, because participants were anonymous to each other and status differences, if existent, were not made salient to them. Thus, Equality moral motives could have been activated in the participants’ minds, especially in the control condition without a manipulation of moral motives (DSG Pilot Experiment). However, it rather seems that Proportionality moral motives dominated the minds of participants in the experiments reported here. Respective analyses of our data revealed that inducing Proportionality moral motives in DSG resulted in decision behavior that is statistically indistinguishable from the behavioral responses in the DSG control condition, without manipulation of moral motives. This finding can be interpreted such that the DSG decision task itself (including the above described “money” reminders) induces Proportionality moral motives or participants came to the experimental laboratory with ‘default’ moral motives pertaining to Proportionality (or both).

More generally, when considering a likely Proportionality framing of any one-shot game experimental setting in which participants are paid for participating (money prime) and in which the task is to allocate proportions of resources or risks (or both) to oneself and to another person, it seems likely that behavioral responses shift toward Proportionality motivated outcomes rather than to “zero solidarity” or purely self-interest motivated outcomes, which are predicted by expected utility theory and game theory (discussed in more detail below).

In summary, proximate characteristics of the experimental decision game itself as well as distant characteristics of the wider experimental context can induce certain moral motives with respective behavioral responses. Behavioral effects of moral motives, whether intentionally stimulated, as in the four experiments reported here, or unintentionally induced and thus often remaining unnoticed, are generally to be expected in many commonly used experimental decision games where participants are confronted with one-shot interpersonal decision situations and can respond with more or less other-regarding decision behavior.

### The Self-Interest Concept in Interpersonal Economic Decision Making

It was pointed out to us by one of the reviewers of the present paper that the Market Pricing and Proportionality constructs are defined within RMT and RRT as a social relational structure and respective moral motives for social coordination with reference to a *socially meaningful* ratio, rate, or proportion. The constructs explicitly exclude any supposition that self-interest or maximization of individual benefit is a defining, necessary, or distinctive feature of the Market Pricing relational model or the Proportionality motive. The idea that self-interest or the maximization of individual benefits is intrinsic to Market Pricing relational models or Proportionality moral motives seems plausible from folk psychology and from economic theory, but it is not part of RMT and RRT.

We concur with the reviewer’s comment which also points out that according to RMT and RRT any of the four relational models and respective moral motives may be behaviorally implemented with more or less self-interested motives. Furthermore, RMT explicitly posits (and RRT implies) that to the extent that behavior toward another person is not regulated by a moral model for coordination, but is instead oriented to using the other person purely instrumentally as a means to individual non-relational ends, the action is governed by an Asocial or Null model ([1], p. 692), which is totally distinct from Market Pricing relational models and respective Proportionality moral motives. 

The results found in the present series of experiments provide empirical support for the RMT and RRT separation of social relational (proportional) rational thinking and doing, on the one side, and self-interest motivated rational thinking and doing on the other side. The solidarity behavior shown by participants in all Proportionality conditions was significantly different from (and higher than) the “zero solidarity” predictions derivable from an Asocial or Null relational model, according to RMT, and also from the “zero solidarity” predictions derivable from expected utility theory and game theory, which both employ the concept of self-interest as their fundamental axiom. Moreover, even with an overall Proportionality framing of the experimental game situations, as was discussed above, plus Proportionality framing and priming conditions, implemented in our series of experiments, participants still give away money to help a stranger, despite the fact that they could keep it without their choice being known. This is strong evidence for the claim of RMT and RRT that Market Pricing relational models and Proportionality moral motives, which evidently guide participants’ behavior in the experimental game paradigms used in the present study, do not constitute purely self-interested maximization of individual benefits. 

## Supporting Information

File S1 Appendices A, B, and C. (The File S1 can be retrieved from PLOS ONE or from www.psy.lmu.de/wirtschaftspsychologie/forschung/working_papers/index.html, see WOP Working Paper 2013/5.) .
(PDF)
