# Supplementary material for: Morals Matter in Economic Games
Source: PLoS One. 2013 Dec 16;8(12):e81558. doi: 10.1371/journal.pone.0081558 (PMC3864843; doi:10.1371/journal.pone.0081558)
Supplement: File S1 — Appendices A, B, and C. (The File S1 can be retrieved from PLOS ONE or from www.psy.lmu.de/wirtschaftspsychologie/forschung/working_papers/index.html, see WOP Working Paper 2013/5.) . (PDF) [file pone.0081558.s001.pdf]

Supporting Information to:

Morals Matter in Economic Games

Felix C. Brodbeck

Katharina G. Kugler

Julia A. M. Reif

Markus A. Maier

Ludwig-Maximilians-Universitaet Muenchen, Munich, Germany

2013

Table of Contents

	Page
Appendix A: The Dyadic Solidarity Game (DSG)	2
Appendix B: The Self-Insurance Game (SIG)	6
Appendix C: Manipulation of Moral Motives	9

APPENDIX A: THE DYADIC SOLIDARITY GAME (DSG)

Description

In the DSG two persons engage in one-shot interpersonal decision making with each other (see Figure A1). In our experiments both receive 10€ to their disposal. Each person can win up to 10€ with a probability of 2/3 (if a dice shows 1, 2, 3, or 4) or lose up to 10€ with a probability of 1/3 (if a dice shows 5 or 6). Before the dice is tossed each person decides whether he or she wants to put money aside, which will be given to the other person in the case of losing. Hence participants can divide the 10€ in two partial amounts (Amount A and Amount B). Each person receives Amount A for himself or herself in case of winning. In case of losing, each person will receive the Amount B put aside by the respective other person. In case the person for who the amount B is put aside wins, the amount B is withhold by the experimenter; thus the gift giving to the other person is unconditional.

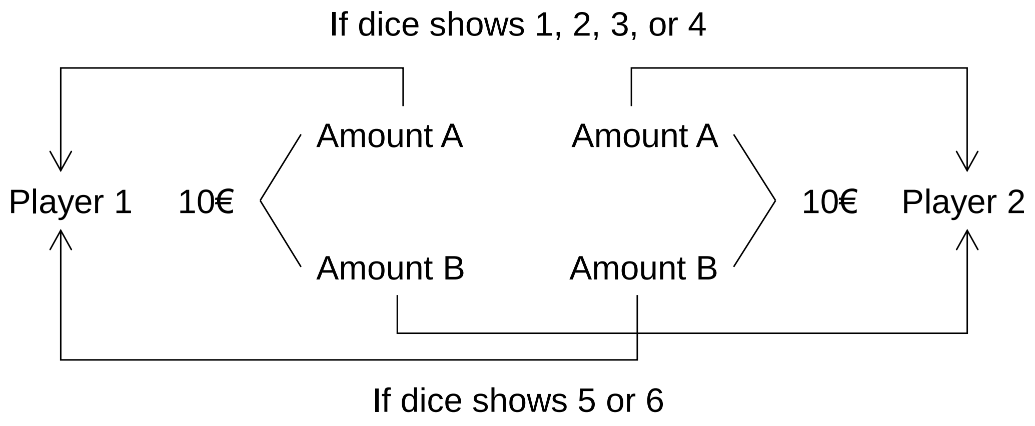

Figure A1. Dyadic Solidarity Game (DSG; two interacting players).

The payoff for each of the two persons of a dyad (Person 1 and Person 2) is depicted below.

$$Payoff_{Person1} = \begin{cases} 10€ - Amount\ B_{Person1} & if\ dice \in \{1, 2, 3, 4\} \\ 0€ + Amount\ B_{Person2} & if\ dice \in \{5, 6\} \end{cases}$$

$$Payoff_{Person2} = \begin{cases} 10€ - Amount\ B_{Person2} & if\ dice \in \{1, 2, 3, 4\} \\ 0€ + Amount\ B_{Person1} & if\ dice \in \{5, 6\} \end{cases}$$

$$Amount\ B \in [0€; 10€]$$

From a classic standard expected utility perspective (i.e., maximizing one's own profit), participants are expected to not allocate any money to Amount B. The expected utility for the economic decision of the two players (Person 1 and Player 2) in the DSG is:

$$E(u_i) = \sum p_x u(x)$$
$$i = \{Person\,1, Person\ 2\}$$
$$u(x) = x$$
$$x = \{Amount\ A_i, Amount\ B_{\neg i}\}$$
$$p_{Amount\,A} = \frac{2}{3}$$
$$p_{Amount\,B} = \frac{1}{3}$$

In our experiments Amount A and Amount B were determined as follows:

$$Amount\ A_i + Amount\ B_i = 10€$$
$$Amount\ A_i \in [0€; 10€]$$

Therefore from a classic standard expected utility perspective a player's optimal choice is:

$$Amount\ A^* = 10€$$

Differences and Similarities Between DSG, SG and Relevant Other Economic Games

The DSG - a two person game - is easier to employ by investigators and easier to understand by participants than the Solidarity Game (SG; [10]) - a three person game. In DSG gift giving is *unconditional*, and gift receiving is conditional upon oneself loosing (i.e., one only receives a gift if the dice shows a 5 or 6). In SG actual gift giving is conditional upon winning and one or two others loosing. Thus, in SG more complex gift-distributions have to be calculated, conditional upon (one or two) others losing, than in DSG. In DSG no distributional considerations among several others need to be made, which reduces cognitive load and the likelihood of confusion.

In several noteworthy respects, SG and DSG are concordantly similar to and distinct from other economic decision making games widely used in the experimental literature for establishing evidence about the expression of other-regarding behavior. SG and DSG are similar to the Dictator Game [9] which is known as a benchmark test for altruism. However, the Dictator Game creates an asymmetric interpersonal situation where only one person is a donor and the other person serves as an (inactive) recipient. The altruism-benchmark of the Dictator Game applies only to participants who take the role of a donor. The SG and DSG are symmetric in the sense that all participants are donors *and* receivers simultaneously. Moreover, SG and DSG both imply a motivational element of trust in reciprocity and mutuality, speaking to the potential expression of moral motives different from pure altruism, which is missing in a one-shot Dictator Game. SG and DSG are also similar to the Public Good Game (for a review see [81]) in that a person can contribute to a common pool. However, they also different, as in Public Good Games often the total amount of the pool can increase depending on the interplay of the players' decisions. In SG and DSG the total amount available does not increase. Instead the donated proportions can be used to mitigate losses of the other person only. This feature of SG and DSG makes them also highly similar to Risk Sharing Games without commitment [59]. Because SG and DSG are one-shot games, they are actually equal to a Risk Sharing Game without commitment with a continuation probability of zero.

DSG Pilot Experiment (Control Condition)

In order to establish a DSG baseline and control condition (no manipulation of moral motives) and to compare the decision making outcomes from DSG with published results from SG [10], [57], 18 participants (sex: 61% female; age: M = 23.94 years, SD = 2.75 years) from engaged in DSG. The laboratory sessions were conducted in the Department of Economics of the Ludwig-Maximilians-Universitaet Muenchen, Munich, Germany. Participants were paid a show-up fee of 4€ in addition to the payoff of the game.

The experiment and its consent procedure were approved by the Research Ethics Committee of the Economics Department at the Ludwig-Maximilians-Universitaet Muenchen, Munich, Germany. Participants provided written consent to the procedures and the standards as well as participants' rights when voluntarily signing up for the panel of the laboratory. Full information about the study was provided to participants prior to the experiment and participants were able to leave the experiment at any time without consequences.

The invitation procedure was analogue to Experiment 3. All 18 participants were seated in cubicles in one room and knew that they interacted with one other participant in the room not known to them. Upon receiving the instructions participants entered their decision (10€ split into Amounts A and B) in a computer (see Figure A1). After submitting a dice was thrown and individual payoffs were shown on the computer screen to each participant. The average Amount B that was given to the other person in case of losing (i.e., the dice showed a 5 or 6) was 2.50€ (*SD* = 1.47€, *Min* = 0€; *Max* = 4€; also see Table 1). This result is inconsistent with the maximum of the expected utility, as the Amount B is significantly greater than 0€ ($t(17) = 7.24$, $p<.001$, 95% CI [1.77, 3.23]). The result is consistent with the results reported by Selten and Ockenfels [10] and Brosig-Koch et al. [57], who present data from an earlier SG study comparing East and West German participants in 1995 and its replication in 2009. In the experiment by Selten and Ockenfels [10] participants were given 10 Deutsche Mark (DM) to their disposal (the study was conducted before the German cash currency was changed from Deutsche Mark [DM] to Euro [€] in January 2002). From the 10DM they gave on average 2.46 DM to the other person in case of losing. For better comparison across the two different currencies, the percentage of the total amount given in DSG was compared to the SG condition "gift to one other person, when one other person was losing" only for the West German group of participants: DSG (2011) = 25.0%, SG (1998) = 24.6%, SG (1995) = 25.8%, SG (2009) = 22.6%.

The data from the DSG Pilot experiment, with appropriate supporting materials and explanations, will be shared upon request.

APPENDIX B: THE SELF-INSURANCE GAME (SIG)

Description

The SIG constitutes a one-shot *solitary* (one player) situation of economic decision making which is identical to the Dyadic Solidarity Game (DSG) in all but one respect: the person interacts with himself or herself (for a visualization of the SIG see Figure B1). Individuals are given 10€ for their disposal and they can win up to 10€ with a probability of 2/3 (if a dice shows 1, 2, 3, or 4) or lose it with a probability of 1/3 (if the dice shows 5 or 6). However, before the dice is tossed each person can choose to put aside some money, which he or she will receive himself or herself in case of losing (i.e., the dice shows 5 or 6). Hence participants can divide the 10€ in two partial amounts: Amount A and Amount B. The person will receive Amount A in case the dice shows 1, 2, 3, or 4 and Amount B in case the dice shows 5 or 6.

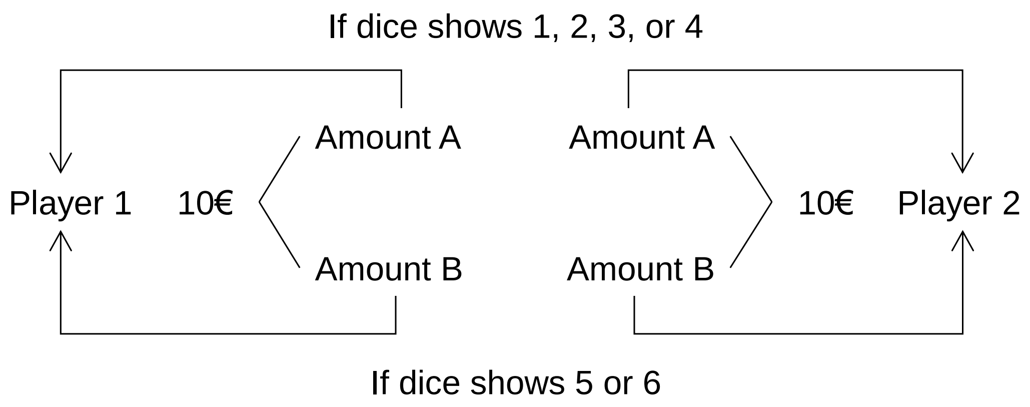

Figure B1. Self-Insurance Game (SIG; two solitary acting players).

The payoff in SIG can be formally described as is shown below.

$$Payoff = \begin{cases} 10€ - Amount\ B & if\ dice \in \{1, 2, 3, 4\} \\ 0€ + Amount\ B & if\ dice \in \{5, 6\} \end{cases}$$
$$Amount\ B \in [0€; 10€]$$

From a classic standard expected utility perspective (i.e., maximizing one's own profit), participants are expected to not allocate any money to Amount B. The expected utility for the economic decision of each player in the SIG is:

$$E(u) = \sum p_x u(x)$$
$$u(x) = x$$
$$x = \{Amount\ A, Amount\ B\}$$
$$p_{Amount\,A} = \frac{2}{3}$$
$$p_{Amount\,B} = \frac{1}{3}$$

In the experiments, which are described in this paper Amount A and Amount B were determined as follows:

$$Amount\ A + Amount\ B = 10€$$
$$Amount\ A \in [0€; 10€]$$

Therefore from a classic standard expected utility perspective a player's optimal choice is:

$$Amount\ A^* = 10€$$

SIG Pilot Experiment

Analogous to the DSG a control condition and baseline (no manipulation of moral motives) was established by conducting a SIG Pilot Experiment. 24 participants (sex: 54% female; age: *M*=23.92 years, *SD*=3.17 years) engaged in SIG. The laboratory sessions took place in the Department of Economics of the Ludwig-Maximilians-Universitaet Muenchen, Munich, Germany. Participants were paid a 4€ show up fee in addition to the payoff of the game.

The experiment and its consent procedure were approved by the Research Ethics Committee of the Economics Department at the Ludwig-Maximilians-Universitaet Muenchen, Munich, Germany. Participants provided written consent to the procedures and the standards as well as participants' rights when voluntarily signing up for the panel of the laboratory. Full

information about the study was provided to participants prior to the experiment and participants were able to leave the experiment at any time without consequences.

The invitation procedure was analogue to Experiment 3. All 24 participants were seated in cubicles in one room during the study. First, participants were given the instructions, second, participants made their decision, third, the dice was tossed and fourth, participants were informed about their payoff. They received the payoff in addition to the 4€ show up fee. The average Amount B that was put aside for oneself in case the dice showed a 5 or 6 was 3.20€ (SD = 1.31€, Min = 0€, Max = 5€; also see Table 1), which is greater than 0€ ($t(23) = 11.99$, p <. 001, 95% CI [2.65, 3.75]) and therefore inconsistent with the maximum of the expected utility.

The data from this study, with appropriate supporting materials and explanations, will be shared upon request.

APPENDIX C: MANIPULATION OF MORAL MOTIVES

Each participant in Experiment 1 and Experiment 3 was presented one of the following two frames at the very beginning of the experimental procedure. Depending on the condition participants received either the Unity frame or the Proportionality frame in order to manipulate their salient relational model. The sentences in italic were only presented to the participants in the DSG and not the participants in the SIG, as they were not interacting with another person.

Unity Frame

Original Instructions in German. “In dieser Studie geht es um das Thema ‘Gemeinwohl in einer Gruppe bzw. Gesellschaft’. Dabei wird untersucht, wie sich Menschen verhalten, wenn sie zum Ziel haben den gemeinschaftlichen Nutzen für ein gesamtes Team, eine Gruppe oder eine ganze Gesellschaft zu maximieren. Wir wollen damit die Frage beantworten, wie in einer Gesellschaft oder Gruppe ein soziales und stabiles Gleichgewicht gefunden werden kann, zu dem alle beitragen und aus dem alle schöpfen können. *Da es in dieser Studie um gemeinschaftliches, kooperatives und soziales Handeln geht, werden Sie in der Experimentalaufgabe mit einem Partner zusammenarbeiten. Dieser wird im Folgenden ‘die andere Person’ genannt.*“

Translated Instructions in English. “This study deals with the topic of ‘common welfare in a group or society’. Thereby it is assessed, how people behave if they aim to maximize the common profit for a whole group or a whole society. We want to answer the question, how people in a group or society can find a social and stable equilibrium, to which everyone is contributing and from which everyone is taking. *As this study is about collective, cooperative and social action, you will have a partner in this experimental task. This person will be named ‘the other person’ hence forth.*”

Proportionality Frame

Original Instructions in German. “In dieser Studie geht es um das Thema "Kosten-Nutzen-Optimierung auf freien Märkten oder an der Börse". Dabei wird untersucht, wie sich Menschen verhalten, wenn sie zum Ziel haben, den individuellen Nutzen für sich selbst zu maximieren und sie mit anderen Personen konkurrieren. Wir wollen damit die Frage beantworten, wie Personen ihren eigenen Gewinn maximieren und in marktwirtschaftlichen

Situationen das Beste für sich herausholen. *Da es in dieser Studie um Konkurrenzsituationen und individuelle Gewinnmaximierung durch Kosten-Nutzen-Kalkulationen geht, werden Sie in der Experimentalaufgabe einen Gegner haben. Dieser wird im Folgenden „die andere Person“ genannt.*“

Translated Instructions in English. “This study deals with the topic of ‘cost-benefit-analysis of free markets or of stock exchange’. Thereby it is assessed, how people behave if they aim to maximize the individual profit for themselves and compete with other persons. We want to answer the question, how people can maximize their own gains and can attain the best results for themselves in situations of free market economy. *As this study is about a situation of competition and individual profit maximization through cost-benefit-analysis, you will have an opponent in this experimental task. This person will be named ‘the other person’ hence forth.*”
